# CIMUVET-survey: Complementary and Integrative Medicine (CIM) use in veterinary practice in Austria and CIM education at universities in Austria, Germany and Switzerland

**DOI:** 10.1371/journal.pone.0327599

**Published:** 2025-07-02

**Authors:** Pia Forster, Annemarie Käsbohrer, Holger Cramer, Michael Frass, Ariane Maeschli, David Martin, Peter Panhofer, Birgit Ursula Stetina, Ursula Wolf, Jürgen Zentek, Petra Weiermayer

**Affiliations:** 1 Clinical Department for Farm Animals and Food System Science, University of Veterinary Medicine, Vienna, Austria; 2 Institute of General Practice and Interprofessional Care, University Hospital Tübingen, Tübingen, Germany; 3 Robert Bosch Center for Integrative Medicine and Health, Bosch Health Campus, Stuttgart, Germany; 4 em. Department of Medicine I, Medical University of Vienna, Vienna, Austria; 5 Scientific Society for Homeopathy (WissHom), Koethen, Germany; 6 Department of Livestock Sciences, Research Institute of Organic Agriculture, Frick, Switzerland; 7 Institute of Integrative Medicine, Department for Medicine, University Witten/Herdecke, Herdecke, Germany; 8 Tübingen Universtity Children’s Hospital, Tübingen University, Tübingen, Germany; 9 Faculty of Medicine, Sigmund Freud Private University Vienna, Vienna, Austria; 10 Faculty of Psychology, Sigmund Freud Private University Vienna, Vienna, Austria; 11 Institute of Complementary and Integrative Medicine, University of Bern, Bern, Switzerland; 12 Institute for Animal Nutrition, Freie Universität Berlin, Berlin, Germany; Borlaug Institute for South Asia-CIMMYT, INDIA

## Abstract

**Introduction:**

Complementary and Integrative Medicine (CIM) are an important component of healthcare worldwide according to the WHO Traditional Medicine Strategy. The Licensing Regulation for Physicians in Germany and the Medical Professions Act in Switzerland stipulate that CIM must be taught as an integral part of the human (DE, CH) or veterinary (CH) degree programme. The aim of this study was to evaluate the status of CIM in veterinary practice in Austria in context with an overview on practice, research and teaching at the universities of human and veterinary medicine in German speaking countries.

**Materials and methods:**

Using a cross-sectional study design, an anonymous questionnaire on the use of CIM in veterinary practice was sent out via the Austrian Veterinary Chamber. Chairs, professorships and institutes, and courses on CIM at universities of human and veterinary medicine were researched online.

**Results:**

Of the 246 voluntary participants, 58.9% reported a positive, 22.4% a negative and 15.4% a neutral attitude towards CIM. Of the livestock veterinarians, 68.9% were familiar with the requirement of the EU Organic Regulation, as were 54.1% of all veterinarians. The integration of CIM into the Vetmeduni Vienna curriculum was rated as very important by 35.8% of participants with at least partial approval by 68.7%. The demand for CIM by patient owners amounted to 83.7% and the use of CIM in animals to 65.9%. At Austrian, German, and Swiss universities, 39 professorships of CIM in human medicine (AT: 2; DE: 32; CH: 5) were identified while in veterinary medicine, seven professorships for animal nutrition and dietetics (AT: 2; DE: 5) were identified.

**Conclusion:**

Based on the results of the CIMUVET study, integrating CIM as in university curricula and hospitals in Switzerland and Germany is a promising future development for Austria. These approaches should follow the WHO Traditional Medicine Strategy 2025–2034.

## Introduction

The World Health Organisation’s (WHO) Traditional Medicine Strategy 2014–2023 as well as the WHO Traditional Medicine Strategy 2025–2034 call for the integration of Complementary and Integrative Medicine (CIM) into national health systems. With this strategy, the WHO aims to ensure that a healthy life and well-being are accessible to all [1]. In veterinary medicine, the national requirements and the requirements of the EU and Swiss organic regulations must be followed. The EU Organic Regulation 2018/848 requires that ‘feed materials of mineral origin […], nutritional additives […], and phytotherapeutic and homeopathic products shall be used in preference to treatment with chemically synthesised allopathic veterinary medicinal products, including antibiotics, provided that their therapeutic effect is effective for the species of animal and for the condition for which the treatment is intended’ [2]. The Swiss Ordinance on Organic Farming and Labelling of Organically Produced Products and Foodstuffs stipulates the similar accordingly [3].

Complementary medicine comprises those medical methods that are not categorised as conventional medicine and are usually used synergistically with conventional medicine [4]. The combination of conventional and complementary medical methods is known as integrative medicine [5,6]. A recent systematic review listed methods summarised with the term complementary medicine [7].

The integration of CIM into the curriculum of veterinary universities is asserted by the American Consensus Guideline on University Education in Integrative Veterinary Medicine, but no similar document exists in Austria and Germany [8]. In Switzerland it is legally anchored in the Medical Professions Act, which is compulsory for universities in both human and veterinary medicine. In Germany the Licensing Regulation for Physicians regulates this requirement for human medicine only [9,10]. Consequently, CIM is a fixed part of the curriculum for human medicine, veterinary medicine and dentistry programmes in Switzerland and for human medicine programmes in Germany [9,10]. CIM is not integrated into the relevant regulations in Austria, for neither human nor veterinary medicine [11,12]. In the veterinary medicine degree programmes in Germany and Austria, only animal nutrition and dietetics as well as botany are permanently integrated into the curriculum. These courses are part of the regular curriculum as mentioned in the Standard Operating Procedure of the European System of Evaluation of Veterinary Training (ESEVT) [13] and not explicitly classified as CIM. Nevertheless, CIM provides, e.g., strategies that contribute to reducing antimicrobial use, preventing or treating infections in both human and veterinary medicine, and may contribute to promoting the health/resilience of humans and animals [14]. In view of the major global challenges such as antibiotic resistance, and based on the requirements of Organic Regulations, the aim of the study was to address the research question ‘What is the status of CIM in veterinary practice in Austria?’ using a cross-sectional design to evaluate the need for possible consequences in university teaching, research and practice [2,15,16]. A survey on CIM was carried out among German veterinarians in the year 2022; to date, no such survey has been conducted among Swiss veterinarians [17]. The results obtained in our CIMUVET study were discussed in context with currently available education on CIM and their sub-disciplines in Austria, Germany and Switzerland in veterinary and human medical universities. The findings should serve to develop future strategies for university teaching, research and practice and to support the achievement of the requirements of the EU Organic Regulation 2018/848 and of the European Green Deal [2,18].

## Materials and methods

### Survey

#### Questionnaire.

For the cross-sectional study a questionnaire (CIMUVET: Complementary and Integrative Medicine Use in VETerinary Medicine) was developed by a multiprofessional team of researchers resulting in 37 questions in six sections (S1A Appendices). To reach all Austrian veterinarians the decision for an online-survey was made using the LimeSurvey survey tool (Community Edition Version 5.6.68, Hamburg, Germany) as provided by the Vetmeduni Vienna taking into account current recommendations to control for order and response bias as far as possible [19–24]. To define the scope of the disciplines to be covered, a comprehensive literature search was conducted in Pubmed regarding the definition of CIM in the field of human and veterinary medicine (S1B Appendix) and experts were consulted with regards to education on CIM provided at their universities. Apart from this, CIM methods included in the questionnaire were cross-checked with the list of veterinary speciality and diploma fields of the Austrian Veterinary Chamber (ÖTK). CIM methods included in the questionnaire had to be named by the definition of CIM according to the findings of the literature search as well as on the list of the ÖTK. The first section of questions consisted of a total of four single or multiple-choice questions regarding the participants’ practice structure. The attitude towards CIM and the use of CIM methods were surveyed in the second section using a single choice, a multiple choice and an open question format. The third section of questions included seven ranking questions with verbal or numerical rating scales, six single-choice questions, two open questions and one multiple-choice question relating to the use of CIM in the veterinary practice. Information on CIM training was collected in the next section on the basis of four single-choice questions and a matrix question. The following group of questions on professional representation comprised a single-choice question and four numerical ranking questions. In addition, this section included the option to provide comments and suggestions regarding the survey in the form of an open question. Finally, the last part of the questionnaire collected demographic data (sex, age, and subject-specific orientation) of the participants.

### Ethics and data protection

The anonymous cross-sectional study was approved by the Vetmeduni Vienna as part of a diploma thesis. The Ethics Committee of the Medical University of Vienna reviewed the ethics application and determined that no submission to the Ethics Committee is required for such an anonymous cross-sectional study. Data protection was assured and informed consent was given in writing (S1A Appendices).

### Sampling

The link to the online survey was sent out to all veterinarians (n = 4137) registered with the ÖTK (as of December 31, 2023) which includes all practicing and some non-practicing veterinarians. This was chosen to control for sampling and non-response bias as much as possible. Participation in the survey was possible via the link provided by email from May 17, 2024 to June 13, 2024. All fully completed questionnaires submitted within the deadline were considered for data analysis and checked for consistency.

### Data analysis

The statistical programme SPSS Version 29.0.2.0 (IBM, Böblingen, Germany) was used for analysis of the collected data. The descriptive statistics included absolute and relative frequencies, and the variability and distribution of the data collected. A completion rate was calculated in relation to the total number of veterinarians in Austria. To test the comparability of the sample with the population of veterinarians, gender and age distribution of the participants in the cross-sectional study were compared with the total number veterinarians registered with the ÖTK. Unlike in the USA/Canada, the degree course in veterinary medicine in Austria concludes with a veterinary diploma entitling to work independently as a veterinarian in all areas [25]. In Austria, a doctorate (Doctorate in Veterinary Medicine (DVM)) or PhD represent a higher university qualification, however, these are not mandatory for practising veterinary medicine. For statistical analysis, two categories were formed to reflect the possible influence of higher university education and higher additional non-university training. The data sets were divided into two categories each: a.) ‘Veterinarians without a PhD or DVM’ and ‘Veterinarians with a PhD or DVM’ as well as b.) ‘Veterinarians with further training’ and ‘Veterinarians without further training’, which summarised postgraduate education, namely Specialist veterinarians (FTÄ: Fachtierärzte), ÖTK diploma holders and other completed training.

For the evaluation of the metric data, an exploratory data analysis was carried out and the statistical significance was calculated using single-factor analyses of variance and the effect size Eta², which was also categorised as low (0.01), medium (0.06) and high (0.14) [26]. For nominal variables, the p-value was determined using the chi-square test and the effect sizes Phi and Cramer’s V were calculated to test for differences between the two categories each [27]. According to Cohen, the effect size was categorised as low (0.1), medium (0.3) and high (0.5) [26]. Normal distribution was tested using the Kolmogorov-Smirnov test. For ordinal variables Kendall’s rank correlation (Kendall’s tau b) was calculated [28]. A p-value of p < 0.05 was set as significant. The answers to open questions were categorised, divided into positive, neutral and negative with regard to the question on attitudes towards CIM and evaluated both quantitatively and qualitatively. To enhance the categorization of the high numbers of diverging answers, the answers to the question on motives to integrate CIM at the Vetmeduni Vienna as well as potential comments and suggestions were assigned to topic-related categories with the help of ChatGPT-4o. Then both the assignment of the answers to the categories and the categories themselves were checked for plausibility by the first and last author due to limitations of ChatGPT-4o in non-English languages, particularly in academic and professional contexts as well as accuracy and potential biases [29,30]. The way ChatGPT-4o is used in our CIMUVET study is comparable to the development of clinical data models with AI, so the same limitations apply, particularly when scaling analyses across increasingly complex and heterogeneous data. Hence, manual review is essential at the current stage of AI process development. Still, the advent of generative artificial intelligence and knowledge graphs heralds a potential next generation in electronic healthcare data utilization [31,32].

### University teaching programme

The first step was to carry out a structured internet search for existing chairs, professorships, institutes, and teaching programmes in CIM and their sub-disciplines on the websites of all universities of human and veterinary medicine in Austria, Germany and Switzerland (DACH region). Curricula, laws, regulations and, where available, catalogues of electives and compulsory electives for the degree programmes offered were taken into account. Information on universities of human medicine were included to gain a further perspective. In the second step, feedback from enquiries to specialists working in CIM were incorporated (S2A Appendix and S2B Appendix). The DACH region was selected for comparison because of the common language and because students and veterinarians/ medical doctors from the three countries, Austria, Germany and Switzerland, are in close professional contact and the organic regulations impose the same requirements with regard to CIM [2,3].

## Results

### CIMUVET survey

There were 246 voluntary participants (6.0% of all 4,137 Austrian veterinarians) in the cross-sectional CIMUVET study. Gender as well as age distribution showed little distortion compared to the total number of veterinarians registered with the ÖTK (shown in Table 1). In comparison to the number of veterinarians with veterinary specialities registered with the ÖTK, a slightly higher proportion of veterinarians with further training participated in the CIMUVET study, i.e., 22.4% vs. 14%. Apart from this, in comparison to the number of veterinarians with PhD or DVM registered with the ÖTK, a slightly lower proportion of veterinarians with PhD or DVM participated in the CIMUVET study, i.e., 31.3% vs. 42.7%. In summary, it can be said that numbers of veterinarians with PhD or DVM or veterinarians with further training showed little distortion compared to the total number of veterinarians registered with the ÖTK (shown in Table 1).

**Table 1:** Comparison of the CIMUVET survey sample with the veterinarians registered with the ÖTK.

		Participants in CIMUVET survey (n = 246)		Veterinarians registered with ÖTK* (n = 4,137)	
**Gender**					
	Female	171	(69.5%)	2,597	(62.8%)
	Male	70	(28.5%)	1,540	(37.2%)
	Diverse	5	(2.0%)	0	
**Age (years)**					
	≤ 30	19	(7.7%)	463	(11.2%)
	31-40	47	(19.1%)	857	(20.7%)
	41-50	78	(31.7%)	924	(22.3%)
	51-60	75	(30.5%)	1,087	(26.3%)
	61-70	23	(9.4%)	582	(14.1%)
	> 70	4	(1.6%)	224	(5.4%)
**Doctorate status: PhD or DVM (Doctorate in Veterinary Medicine)**					
	Yes	77	(31.3%)	1765	(42.7%)
	No	169	(68.7%)	2372	(57.3%)
**Further training: specialist veterinarians (FTÄ), ÖTK diploma holders and veterinarians with other completed training**					
	Yes	55	(22.4%)	578	(14.0%)^#^
	No	191	(77.6%)	3559	(86.0%)^#^

ÖTK: Österreichische Tierärztekammer – Austrian Veterinary Chamber (*as of December 31, 2023); ^#^ Numbers of veterinarians with veterinary specialties and ÖTK diploma holders are provided by ÖTK (as of December 31, 2023).

An overview of the answers received, broken down for a.) veterinarians with and without a PhD or DVM, and b.) veterinarians with and without further training which summarised Specialist veterinarians (FTÄ), ÖTK diploma holders and veterinarians with other completed training, was created with details of the p-values and effect sizes for each comparison (veterinarians with and without PhD or DVM respectively with and without further training) separately (shown in Table 2).

**Table 2:** CIMUVET questionnaire: Comparison of responses of two different groups of veterinarians: a.) veterinarians with PhD or DVM (Doctorate in Veterinary Medicine) and veterinarians without PhD or DVM (middle two columns) and comparison of responses of b.) veterinarians with further training and veterinarians without further training (last two columns).

No.	Question	Comparison of veterinarians with (n = 77) PhD or DVM and without (n = 169) PhD or DVM		Comparison of veterinarians with (n = 55) further training and without (n = 191) further training	
		p-value	Phi/ V/ Eta^2^	p-value	Phi/ V/ Eta^2^
5.1	Use of acupuncture and neural therapy	0.04*	Phi = −0.13	0.13	Phi = −0.10
5.2	Use of chiropractic	0.01*	Phi = −0.17	0.39	Phi = −0.06
5.3	Use of nutrition and dietetics	0.78	Phi = 0.02	0.20	Phi = 0.08
5.4	Use of nutritional advice for small animals	0.63	Phi = 0.03	0.04*	Phi = 0.13
5.5	Use of homeopathy	0.47	Phi = −0.05	0.21	Phi = −0.08
5.6	Use of physiotherapy and rehabilitation medicine	0.39	Phi = −0.06	0.21	Phi = −0.08
5.7	Use of phytotherapy	0.64	Phi = 0.03	0.20	Phi = −0.08
5.8	Use of other methods	0.0004*	Phi = −0.23	0.25	Phi = −0.07
5.9	No use of CIM disciplines	0.50	Phi = −0.04	0.57	Phi = 0.04
*6*	*Use of other CIM disciplines (free text)*	–	–	–	–
7	Attitude towards CIM	0.03*	V = 0.19	0.27	V = 0.13
8	Relevance in practice	0.03*	Eta^2^ = 0.02	0.001*	Eta^2^ = 0.042
9	Use of CIM	0.001*	V = 0.32	0.003*	V = 0.29
10	Frequency with which patient owners mention CIM	0.16	Kendall-Tau-b = −0.08	0.03*	Kendall-Tau-b = −0.13
11	Use of complementary medicine by patient owners	0.27	Eta² = 0.01	0.89	Eta² = 0.0001
12	CIM in veterinary hospitals	0.28	Eta² = 0.005	0.08	Eta² = 0.01
13	Recognition of CIM methods by a veterinary specialty certification/diploma	0.62	Eta² = 0.001	0.007*	Eta² = 0.03
14	Number of veterinary specialties certificates/diplomas sufficient?	0.14	V = 0.13	0.0003*	V = 0.26
*15*	*New veterinary specialties certificates/diplomas (free text)*	–	–	–	–
16	Requirement of the EU Organic Regulation 2018/848	0.04*	Phi = −0.13	0.05	Phi = −0.12
17	Integration into the curriculum of the Vetmeduni Vienna	0.15	Eta² = 0.01	0.047*	Eta² = 0.02
18	Integration – in which form?	0.57	V = 0.11	0.29	V = 0.15
*19*	*Why is integration important? (free text)*	–	–	–	–
20	Demand in practice	0.08	Eta² = 0.01	0.002*	Eta² = 0.04
21	Patients treated with CIM in % per week	0.01*	Eta² = 0.02	0.002*	Eta² = 0.04
*22*	*Diseases treated with CIM (free text)*	–	–	–	–
23	Most common disease/ indication	0.047*	V = 0.36	0.76	V = 0.24
24	Frequency of participation in training courses	0.07	Kendall-Tau-b = −0.11	0.07	Kendall-Tau-b = −0.10
25.1	Training via specialist journals	0.15	Kendall-Tau-b = −0.10	0.05	Kendall-Tau-b = −0.12
25.2	Training via webinars	0.10	Kendall-Tau-b = −0.11	0.005*	Kendall-Tau-b = −0.17
25.3	Training via training events	0.10	Kendall-Tau-b = −0.11	0.00001*	Kendall-Tau-b = −0.26
25.4	Training via congresses	0.07	Kendall-Tau-b = −0.13	0.0001*	Kendall-Tau-b = −0.26
26	Training by the Chamber of Veterinarians	0.94	V = 0.02	0.40	V = 0.09
27.1	Through training providers selected by ÖTK	0.17	Phi = 0.13	0.69	Phi = −0.04
27.2	Through working groups of veterinarians interested in CIM	0.06	Phi = −0.17	0.61	Phi = −0.05
28	Training by the training associations	0.67	V = 0.06	0.09	V = 0.14
29	Importance of diplomas for your own practice	0.88	Eta² = 0.0001	0.00003*	Eta² = 0.09
30	Is training sufficient for practice?	0.89	Eta² = 0.0001	0.27	Eta² = 0.006
31	Coverage of CIM therapies by animal insurance companies	0.97	Eta² = 0.00001	0.047*	Eta² = 0.02
32	Relevance of cost coverage for animal owners	0.19	Eta² = 0.007	0.70	Eta² = 0.001
33	Use of CIM under veterinary responsibility	0.80	V = 0.04	0.44	V = 0.08
*34*	*Comments/ suggestions (free text)*	–	–	–	–

The data sets were divided into two categories: a.) ‘Veterinarians without a PhD or doctorate (DVM)’ and ‘Veterinarians with a PhD or DVM’, and b.) ‘Veterinarians without further training’ and ‘Veterinarians with further training’ which summarised Specialist veterinarians (FTÄ), ÖTK diploma holders and veterinarians with other completed training, statistically significant p-values (< 0.05) are marked with *. Veterinarians with and without a PhD or DVM, and the veterinarians with and without further training were compared separately. Details of the p-values and effect sizes were given for each comparison (veterinarians with and without PhD or DVM (middle two columns) respectively with and without further training (last two columns)) separately.

**Fig 1:**
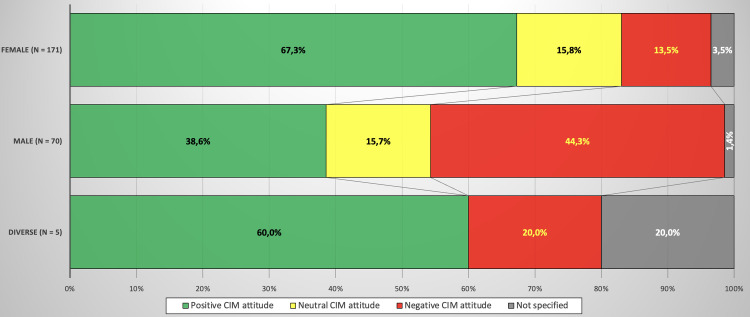
Attitudes towards CIM in relation to gender.

The CIMUVET survey showed that 145 veterinarians (58.9%) report a positive attitude, 55 (22.4%) a negative one and 38 (15.4%) present themselves neutral towards CIM while 8 (3,3%) chose the answer option ‘no answer’. Within the group, it was shown that women are significantly more favourable towards CIM than men (67.3% vs. 38.6%) with a small to medium effect size (p = 0.000007; V = 0.26) (shown in Fig 1).

Furthermore, the data showed with statistical significance with a small to medium effect size (p = 0.03; V = 0.19) that veterinarians with a PhD or DVM, among others, answered the question about their attitude towards CIM more decidedly, i.e., the majority chose either ‘positive’ or ‘negative’ (65.0% vs. 26.0%). The answer option ‘neutral’ was selected more frequently by veterinarians without a PhD or DVM compared to those with a PhD or DVM (20.1% vs. 5.2%) while three veterinarians with a PhD or DVM (3.9%) chose the answer option ‘no answer’.

**Fig 2:**
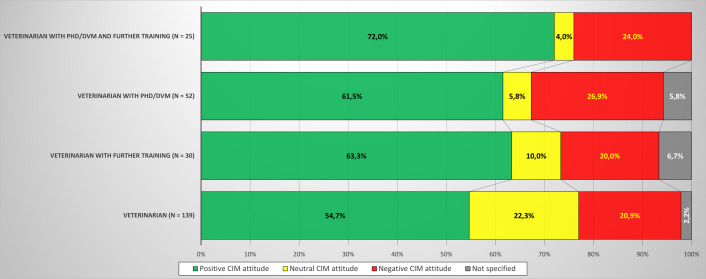
Attitudes towards CIM in relation to PhD or DVM and/or further training.

Over two fifths of the CIMUVET cohort (43.5%; n = 107) had a higher graduate degree (PhD/DVM) and/or further training. Generally, respondents with a higher graduate degree and/or further training had a more definite attitude towards CIM than veterinarians without a higher graduate degree (PhD/DVM) or further training (neutral attitude towards CIM: ≤ 10% versus 22.3%) (Fig 2).

The subjective relevance of CIM for their own practice showed a statistically significant difference between veterinarians with and without a PhD or DVM with a small to medium effect size in favour of veterinarians with PhD or DVM (39.61 vs. 29.5; p = 0.03; Eta^2^ = 0.02). In line with this, on average, veterinarians with further training considered CIM methods to be more relevant than those without further training with a medium effect size (46.0 vs. 28.8; p = 0.001; Eta² = 0.04). This can be summarised as a higher subjective relevance which might also explain the higher practical relevance (= use) of CIM for veterinarians with a PhD or DVM (p = 0.001; V = 0.32) and for veterinarians with further training (p = 0.003; V = 0.29).

When asked about the percentage of patients treated with CIM per week, 79 participants (32.1%) selected the answer option ‘none/sporadic’, 86 (35.0%) selected one to 50.0% and 76 (30.9%) selected more than 50.0% of patients per week, resulting in a positive response rate of 65.9%. Five participants (2.0%) chose the answer option ‘no response’.

The demand for CIM in the practice, was rated ‘10 - very high’ by 53 veterinarians (21.5%) and 206 (83.7%) responded that there was demand. The subjective experience of the demand is significantly more frequent among veterinarians with further training (6.38 vs. 4.76; p = 0.002; Eta² = 0.04). Six participants (2.4%) selected the answer option ‘no response’. The higher level of training in CIM might explain the greater demand for CIM in the practice of veterinarians with further training and thus represents a competitive advantage over veterinarians without further training in CIM. The importance of offering CIM methods in veterinary hospitals was rated ‘10 - very important’ most frequently (32.5%; n = 80). The answer options six to ten were selected by 127 veterinarians (51.6%), answer options two to five were chosen by 45 veterinarians (18.3%) and the answer option ‘1 - not important’ was selected by 67 veterinarians (27.2%), resulting in a total of 172 veterinarians (69.9%) being in favour of offering methods of CIM in veterinary hospitals. Seven respondents (2.9%) selected the ‘no response’ option. This result is also supported by the demand for cooperation between conventional and complementary medicine expressed by several veterinarians in the free text, as can be seen in this example: ‘The individual veterinary speciality fields must work together and not against each other! Medicine cannot function without complementary medicine, but neither can complementary medicine without conventional medicine.’

Additionally, the data showed that 133 Austrian veterinarians (54.1%) stated that they are aware of the requirement of the EU Organic Regulation 2018/848 for preferential use of phytotherapy, feed materials of mineral origin, nutritional additives and homeopathy under the conditions specified therein [2]. The comparison of veterinarians with and without a PhD or DVM revealed a significant difference with a small effect size (p = 0.04; Phi = −0.13; n = 246): 49 (63.6%) of veterinarians with a PhD or DVM were aware of these requirements, while this is true only for 84 (49.7%) veterinarians without a PhD or DVM. In the question on subject-specific orientation, 180 participants selected farm animals (poultry, pigs or ruminants), of which 124 (68.9%) were aware of the requirement of EU Organic Regulation 2018/848. The higher level of knowledge of legal principles, in this case relating to the EU Organic Regulation, among veterinarians with a PhD or DVM, and livestock veterinarians, could be attributed to their further university education and awareness of new legal obligations for those with PhD or DVM, and to the increased relevance to their practice for large animal veterinarians.

The importance of the integration of CIM methods into the Vetmeduni curriculum was rated ‘10 - very important’ most frequently (35.8%; n = 82). Overall, 131 participants (17.5%) considered the integration to be important (answer options six to nine), 38 participants (15.5%) considered it less important (answer options two to five) and 75 participants (30.5%) considered it not important, resulting in 169 participants (68.7%) being in favour of integrating CIM into the curriculum at Vetmeduni Vienna. Two participants (0.8%) chose the answer option ‘no response’. The thirteen comments on the topic of integrating CIM into university teaching confirm this result. The following comment is cited as an example: ‘Integration into university teaching and research is necessary, also in line with the One Health approach!’

### University teaching programme

#### Veterinary medicine.

The internet research on university teaching programmes revealed that in the veterinary field animal nutrition and dietetics is a fixed part of the veterinary medicine curriculum in Austria, Germany, and Switzerland. In Austria, in addition to botany, which is permanently integrated into the curriculum, the Vetmeduni Vienna, the only university of Veterinary Medicine in Austria, offered various CIM electives and compulsory electives in the fields of acupuncture and neural therapy, nutrition and dietetics, phytotherapy, physical medicine and rehabilitation [11]. In Germany, the range of CIM courses varied from university to university, with the exception of the courses ‘Animal Nutrition and Feed Science’ and ‘Botany of Feed, Poisonous and Medicinal Plants’, which are prescribed by the Licensing Regulation for Veterinarians and are permanently integrated into the curriculum [33]. For example, the Justus-Liebig-University of Gießen also included ‘Homeopathy, Phytotherapy and Anthroposophic Therapy’ in the form of a lecture in the curriculum [34]. In Switzerland, CIM and its sub-disciplines are an integral part of veterinary medicine studies, as required by the Medical Professions Act [9], with a total of eight mandatory lectures and some further CIM lectures.

In terms of professorships, there are five professorships in animal nutrition and dietetics identified in Germany, and two professorships in animal nutrition and dietetics in Austria [11,34–38] (S2A Appendix).

#### Human medicine.

In Germany, the Licensing Regulation for Physicians prescribes that a cross-sectional area of CIM and its sub-disciplines are to be permanently integrated into the curriculum of medical schools as cross-sectional area number 12 [10]. As in veterinary medicine, in Switzerland, CIM and its sub-disciplines are an integral part of human medicine studies, as required by the Medical Professions Act [9].

In terms of the number of chairs, in human medicine in Austria, Germany and Switzerland, there are a total of nine chairs for CIM or its sub-disciplines (six in Germany, two in Switzerland and one in Austria) [39–45]. Furthermore, it was found that Germany, in comparison with Austria and Switzerland, has a high number of professorships (n = 26) in CIM or its sub-disciplines in human medicine, while three professorships were found in Switzerland and one in Austria [41–43,46–57] (S2B Appendix).

## Discussion

Hoenders et al. 2024 have addressed the status of conventional medicine, the integration of traditional and complementary medicine, the current role of integrative medicine, and the outstanding demands of the WHO’s 2014–2023 Traditional Medicine Strategy from 2013, in order to be able to contribute to the new 10-year WHO Traditional Medicine Strategy (2025–2034) for CIM, which is scheduled to be published in 2025. In this article, a distinction is made between conventional and non-conventional medicine, the latter comprising a broader health approach, integrative medicine, complementary medicine and traditional medicine [1]. According to the National Center for Complementary and Integrative Health (NCCIH), methods that do not correspond to conventional medicine and are used to supplement conventional therapy are considered complementary medicine [5]. The Consensus Guideline for an integrative veterinary curriculum at US veterinary medical universities defines integrative veterinary medicine as a combination of complementary therapies with conventional medical treatment based on the best available evidence; this is in line with the integrative medicine definition by the NCCIH [5,8]. The term ‘alternative medicine’ can be considered obsolete in light of the definition of ‘integrative medicine’.

Since the definitions of CIM change dynamically over time and may vary depending on the location, an operational definition with an overview of the methods that can be considered as complementary medicine was created in the systematic review by Ng et al. 2022, which is also referenced by Cochrane Complementary Medicine [7,58,59]. The operational definition according to Ng et al. 2022, like four other systematic reviews, two position papers, five expert opinions and one search string, included all complementary medical methods mentioned in our questionnaire (acupuncture and neural therapy, chiropractic, nutrition and dietetics, homeopathy, physiotherapy and rehabilitation medicine, and phytotherapy) [7,8,58,60–69]. In summary, all complementary medicine methods included in the questionnaire were mentioned ten times in 12 articles on complementary medicine in human medicine (except for neural therapy), six times in nine articles on integrative medicine in human medicine (except for neural therapy) and two times in three articles on CIM in veterinary medicine [7,8,58,60–69]. In this context, however, it must be taken into account that the differing mentions of CIM methods is also due to the fact that some CIM subdisciplines can be used in the same way as conventional medical treatment, depending on the situation, such as nutrition and dietetics. Homberg et al. 2022 categorised those methods as complementary medical treatment options that either prevent diseases or are used synergistically with conventional medical therapy for existing diseases [1]. According to Hoenders et al. 2024 or the Academic Consortium for Integrative Medicine, integrative medicine focuses on the whole individual, is based on scientific evidence and uses all appropriate therapeutic and broader health approaches to achieve optimal health and healing [1,70]. Integrative medicine is based not only on the concept of pathogenesis, which underlies conventional medicine, but also on the concept of salutogenesis, the concept of maintaining and restoring health. This means that stress reduction and broader health approaches, such as nutrition and dietetics, can be used as non-conventional medical prophylaxis and therapy to strengthen resilience and thus the immune system [64,71,72].

In the CIMUVET survey the gender and age distribution of the cross-sectional study participants was comparable to the total number of veterinarians registered with the ÖTK, as was the number of veterinarians with a PhD or DVM or further training. The response rate of 6.0% is to be expected for such a questionnaire-based study and comparable to other cross-sectional studies in human and veterinary medicine [17,73–76]. The systematic reviews on cross-sectional studies by Frass et al. 2012, Armano et al. 2025 and Eardley et al. 2012 in medical personnel and in the total population worldwide [63,77] and in the general population in the EU [60,77], found a similar gender-dependent difference in attitudes towards CIM as seen in the cross-sectional CIMUVET study, where female veterinarians were more positive towards CIM than male veterinarians. The hypothesis of women generally exhibiting a stronger preference for holistic approaches was postulated [77]. Overall, in our cross-sectional study veterinarians showed a similarly positive attitude towards CIM as the paediatric clinicians and/or researchers worldwide (58.9% vs. 62.0%) [76]. The U-shaped distribution of the majority of the metric data collected generally in the CIMUVET study indicates that the participants have a strong opinion for or against with respect to a topic. When interpreting this result, it needs to be taken into account that knowledge of the topic is shown to be a major driver of survey participation in general [78]. Apart from this the current, often non-scientific debate about some CIM disciplines could also be a causal factor in this phenomenon and might be subject to further qualitative research [79].

The frequency of use of CIM in veterinary practice (65.9%) is in line with the ranges given by Frass et al. 2012 and Eardley et al. 2012 for the frequency of use of CIM among healthcare professionals and in the general population worldwide [63] and in the general population in the EU respectively [60]. According to Stanossek et al. 2022, the frequency of CIM use in veterinary practice was higher in Germany (85.4%) than in the cross-sectional CIMUVET study conducted in Austria [17]. According to our results, the existing demand for CIM (83.7%) is even higher than the frequency of its use. These results are comparable to those of a cross-sectional study conducted in 2019, in which 97.0% of Swiss paediatricians were asked about CIM treatment methods [80].

It was found that 69.9% of veterinarians in Austria tend to favour the integration of CIM into services offered by veterinary hospitals. In human medicine, a total of 15 institutes and centres with various CIM focuses have been established in Austria, Germany and Switzerland [57][42–49,[41]^51^–^56^]. This development is in line with the wishes and demands of patients in the fields of oncology, cardiology, gynaecology, surgery and gastrointestinal patients at university hospitals [81]. The development of university centres, hospitals and institutes offering CIM methods is desirable in the interests of animal patients and also justified by the high demand from pet owners in the field of veterinary medicine.

In Austria, as in the entire European Union, the requirements of the EU Organic Regulation 2018/848 are to be complied within veterinary medicine for organic production, while the European Green Deal requires increasing the number of organic farms in the European Union (EU) from 8% to 25% by 2030 [2,18]. In the cross-sectional CIMUVET study, 54.1% of Austrian veterinarians were aware of this requirement of the EU Organic Regulation 2018/848, and as many as 68.9% of the participants with a specialist focus on livestock (poultry, pigs or ruminants) [2]. The higher level of knowledge of legal principles, in this case relating to the EU Organic Regulation, among veterinarians with special focus on livestock could indicate that this content should be integrated more extensively into the curriculum of the diploma programme for all students. These data are in line with a recent cross-sectional study conducted among Austrian veterinarians, with livestock veterinarians having the highest level of knowledge regarding the prudent use of antibiotics [74].

The higher relevance of CIM for one’s own practice, the higher use of CIM, the higher demand for CIM, the greater number of patients treated per week with CIM, to name just a few examples, when comparing veterinarians with further training with those without further training – all statistically significant in favour of veterinarians with further training – appear to be logically explained: Further CIM training provides veterinarians with the necessary knowledge and skills to treat patients with CIM more professionally. The greater relevance of CIM for their own practice, the higher frequency of CIM use, the greater number of patients treated weekly with CIM and the higher awareness of the requirement of the EU Organic Regulation 2018/848 for preferential use of phytotherapy, feed materials of mineral origin, nutritional additives and homeopathy under the conditions specified therein, to name just a few examples, when comparing veterinarians with and without a PhD or DVM – all statistically significant in favour of veterinarians with PhD or DVM – could be the subject of further studies as higher scientific/academic qualification seems to be a predisposing factor with positive influence on relevance, use and awareness of CIM. This hypothesis is supported by studies showing that higher education is a predictor of CIM use [63,82,83]. In summary, veterinarians with further qualifications, whether through academic qualifications or further training, seem to look into CIM more closely and may also see more areas of application.

According to the definition of its founder David Sackett and Cochrane Germany, modern evidence-based medicine (EbM) is based on three pillars: individual clinical experience, the values of patients, and the current state of clinical research [84,85]. The aim of EbM is to integrate the experience of the clinician, the values of the patient, and the best available scientific information to guide decision-making about clinical management. All CIM methods mentioned in the questionnaire might be considered in the context of evidence-based medicine as they are based on those three pillars. This should be supported by adequate research and training.

Based on the cross-sectional CIMUVET study, it was found that 68.8% of the participants were at least partially in favour of incorporating CIM into the curriculum at the University of Veterinary Medicine Vienna. In a corresponding cross-sectional study in human medicine in Austria, Germany and Switzerland, 84.0% of the participating department directors at medical schools were in favour of integrating CIM methods into the research and teaching at medical universities and only 34.0% stated that CIM was already integrated into the curriculum [86]. Further on, surgery clinicians and researchers show strong interest in more CIM education and research [87]. This requirement is already clearly stated by the American Consensus Guideline on university education in integrative veterinary medicine and is legally anchored in Switzerland by the Medical Professions Act and in the Licensing Regulation for Physicians in Germany [8–10]. In Austria, there are no legal requirements in this regard. Based on the aspects mentioned above, the integration of education of CIM at universities of human/veterinary medicine should correspond to the demand. The GMA committee on integrative medicine and pluralism of perspectives on natural medicinal products, CIM in medical education, together with the forum of university working groups, has published a position paper [64] based on best practice examples such as University Hospital of Tübingen and University of Witten/Herdecke among others [40–43,46,51,54–56,88].

The strength of the current study lies in the identification of the Austrian veterinary profession’s status and view of, as well as motives, needs and prerequisites for the integration of CIM disciplines in practice, research and teaching at the Vetmeduni Vienna. This is contrasted with best-practice examples of courses, chairs, professors, university institutes and centres for CIM and their sub-disciplines, especially at human medicine universities. The low level of variation of respondents compared to the population of Austrian veterinarians should also be positively emphasised. The limitations include: the lack of questions regarding the size of the practice and practice ownership in the survey to assure anonymity, the impossibility of making statements for the individual disciplines (regarding use, demand, etc.) due to the small sample size, and the possibility of an incomplete list of university teaching offered in Austria, Germany and Switzerland, due to access restrictions to university websites as well as no implementation of a cross-sectional study on courses, chairs, professors, university institutes and centres for CIM and their sub-disciplines. Since the study was conducted in only one country, this must be considered a limitation of the study in terms of the generalisability of the data. Replicating the study in multiple countries would help validate these results and provide a broader perspective. Apart from this, the reliance on self-reported data introduces the possibility of response bias. Participants may have overreported or underreported their CIM usage or attitudes due to recall bias.

Out of 52 universities for human medicine in the three German-speaking countries a total of nine chairs for CIM and its sub-disciplines (six out of 38 universities in Germany, two out of eight in Switzerland, and one out of six in Austria) were identified [39–45]. In Germany, in line with the higher number of universities, a higher number of professorships (n = 26) in CIM and its sub-disciplines in human medicine were counted. In comparison, three professorships have been established in Switzerland and one in Austria [57][41–43,46–56]. Austria and Switzerland are roughly comparable in terms of population, while Germany has a population that is about nine times larger. In veterinary medicine, five professorships in animal nutrition and dietetics were identified in Germany and two in Austria [11,34–38]. The Licensing Regulation for Physicians in Germany and the Swiss Medical Professions Act stipulate that CIM must be taught as a fixed component of human (DE, CH) and veterinary (CH) medical studies. In Germany, CIM is integrated into human medicine studies in the form of a cross-sectional area for rehabilitation, physical therapy and natural healing methods in accordance with the Licensing Regulation for Physicians [10]. As CIM is not integrated into the relevant regulations in Austria, neither for human nor veterinary medicine, with the exception of botany and nutrition and dietetics in the latter field, a need for an update of the existing regulations for human and veterinary medicine in Austria is identified [11,12]. Integrating CIM education into veterinary medical curricula can equip veterinarians with the knowledge and skills needed to engage in informed discussions with patient owners. Such initiatives could also support interdisciplinary collaboration based on scientific evidence and experience, enabling the development of holistic treatment plans that align with patient owner preferences in accordance with the three pillars of evidence-based medicine [85].

## Conclusion

Based on the results of the cross-sectional CIMUVET study, it can be summarized that both the legal and university anchoring of CIM and patient care in university hospitals or centres with CIM in Switzerland and/or Germany represents a desirable future development in Austria. Our findings underscore the need for targeted educational initiatives to bridge the gap between patient owner preferences and veterinarians’ knowledge of CIM. In general, the field of CIM appears to have a promising future in view of major global challenges such as the problem of antibiotic resistance. According to Hoenders et al. 2024, education in CIM in the university sector as a whole should urgently be further expanded as part of the WHO Traditional Medicine Strategy 2025–2034 for CIM. Such programmes could enable veterinarians to engage in informed discussions with patient owners about CIM, fostering a more collaborative and integrative medical approach. In promotion of the one health concept, the potential of CIM in effective prevention and therapy of infectious diseases, requires exploration. This would also support the urgent EU demands for the reduction of antibiotics. In conclusion, this research highlights the critical role of education, communication, and policy development in optimizing CIM use in veterinary medicine in Austria.

## Supporting information

S1A Appendix Questionnaire. Use of complementary medicine in veterinary practice in Austria.
(PDF)

S1B Appendix Literature search. Literature research Definition of terms ‘complementary and integrative medicine’ in human and veterinary medicine.
(PDF)

S2A Appendix University teaching programme in veterinary medicine. Overview of university courses in veterinary medicine in Austria, Germany and Switzerland.
(PDF)

S2B Appendix University teaching programme in human medicine. Overview of university courses in human medicine in Austria, Germany and Switzerland.
(PDF)
