# Supplementary material for: CIMUVET-survey: Complementary and Integrative Medicine (CIM) use in veterinary practice in Austria and CIM education at universities in Austria, Germany and Switzerland
Source: PLoS One. 2025 Jul 2;20(7):e0327599. doi: 10.1371/journal.pone.0327599 (PMC12221077; doi:10.1371/journal.pone.0327599)
Supplement: S1A Appendix — Use of complementary medicine in veterinary practice in Austria. (PDF) [file pone.0327599.s001.pdf]

Use of complementary medicine in the veterinary practice in Austria

Dear ,

Thank you very much for your interest in this scientific study as part of my diploma thesis!

This questionnaire deals with the use of complementary medicine in veterinary practice in Austria. This survey was created in the course of writing a scientific diploma thesis at the University of Veterinary Medicine, Vienna (Vetmeduni Vienna). Participation in the study is voluntary and can be terminated at any time.

In this survey, we investigate the following questions:

The use of complementary medicine in veterinary practice, university and postgraduate education and training as well as professional representation in the field of complementary medicine.

In this study, we ask you to complete a questionnaire after reading and acknowledging the privacy policy. The survey should take about 10 - 15 minutes to complete. We kindly ask you not to forward the survey.

The study is supervised by the Center for Food Safety and Public Veterinary Medicine of the Vetmeduni Vienna, Veterinärplatz 1, 1210 Vienna.

If you have any questions, please contact Pia Forster at11836451@students.vetmeduni.ac.at (mailto: 11836451@students.vetmeduni.ac.at).

This is an anonymous survey!

The protection of your personal data is very important to us in this survey. Therefore, no personal information about you will be stored in the survey responses!
We also ask you not to disclose any personal data such as your name, e-mail address, telephone number or home address. This term refers all data relating to an identified or identifiable natural person.

If you have used an access code for this survey, the software manufacturer of LimeSurvey ensures that the access key is not stored together with the data. It is kept in a separate table and is only updated to record whether you have completed this survey or not. Furthermore, the software manufacturer of assures that there is no way to merge the access codes with the survey results.

When working with a professional online tool such as LimeSurvey, the IP addresses of participants are not stored and the logging of IP addresses is technically suppressed from the outset in accordance with the manufacturers of LimeSurvey in order to guarantee the data protection of the anonymous survey and a high technical security standard. Logging is usually understood to mean the processing chain consisting of the receipt, storage and possible evaluation of data.

This survey contains 37 questions.

Practice structure

Your veterinary activity is: *

Please select the answers that apply:
Please select all applicable answers:

- [ ] Full-time / in full-time or part-time
- [ ] Extraprofessional
- [ ] Employed
- [ ] Self-employed
- [ ] Not working as a veterinarian

What is your professional training? *

Please select the answers that apply:
Please select all applicable answers:

- [ ] Veterinarian
- [ ] Veterinarian plus PhD or doctorate
- [ ] ÖTK diploma holder
- [ ] Specialist veterinarian
- [ ] Diplomate of the European College
- [ ] Professor
- [ ] Other: ____________

Your veterinary specialty field *According to the Austrian Veterinary Chamber (ÖTK) *

Only answer this question if the following conditions are met:
Answer was 'Specialist veterinarian' for question '[G01Q03]' (What is your professional training?)

Please select the answers that apply:
Please select all applicable answers:

- ☐ Acupuncture and neural therapy
- ☐ Anatomy
- ☐ Bees
- ☐ Chiropractic
- ☐ Dermatology
- ☐ Nutrition and dietetics
- ☐ Poultry
- ☐ Homeopathy
- ☐ Small animals
- ☐ Clinical laboratory diagnostics
- ☐ Laboratory animal science
- ☐ Food Sciences
- ☐ Pathology
- ☐ Horses
- ☐ Pharmacology and toxicology
- ☐ Physiotherapy and rehabilitation medicine
- ☐ Pigs
- ☐ Animal husbandry, animal welfare and behavioral medicine
- ☐ Animal breeding
- ☐ Ruminants
- ☐ Wildlife and zoo animals
- ☐ Other: ____________

Your veterinary diploma
* according to the Austrian Veterinary Chamber (ÖTK) *

Only answer this question if the following conditions are met:
Answer was 'ÖTK diploma holder' for question '[G01Q03]' (What is your professional training?)

Please choose the answers that apply:
Please select all applicable answers:

- [ ] Diseases birds, reptiles and small pets
- [ ] Nutritional advice for small animals
- [ ] Farm animal medicine
- [ ] Small animal oncology
- [ ] Equine dentistry
- [ ] Veterinary phytotherapy
- [ ] Dental and oral surgery for small animals and pets
- [ ] Other: ____________

Do you use complementary medical methods and what is your attitude towards them?

Which of the following complementary medicine methods do you use?

List according to Johns Hopkins School of Medicine reduced to those methods that, according to the Austrian Veterinary Chamber (ÖTK) represent specialist veterinary fields or require a diploma.

Please select the answers that apply:

Please select all applicable answers:

- ☐ Acupuncture and neural therapy
- ☐ Chiropractic
- ☐ Nutrition and dietetics
- ☐ Nutritional advice for small animals
- ☐ Homeopathy
- ☐ Physiotherapy and rehabilitation medicine
- ☐ Veterinary phytotherapy
- ☐ Other
- ☐ None

www.hopkinsmedicine.org/health/wellness-and-prevention/types-of-https://complementary-and-alternative-medicine (https://www.hopkinsmedicine.org/health/wellness-and-prevention/types-of-complementary-and-alternative-medicine)

I also use the following complementary medical methods: *

Only answer this question if the following conditions are met:
Answer was 'Other' for question '[G01Q06]' (Which of the following complementary medicine methods do you use? *List according to Johns Hopkins School of Medicine reduced to those methods that, according to the Austrian Veterinary Chamber (ÖTK) represent specialist veterinary fields or require a diploma).
Please enter your answer here:

What is your attitude towards complementary medicine? *

Please select one of the following answers:
Please select only one of the following answers:

- ◯ Positive
- ◯ Neutral
- ◯ Negative
- ◯ Not specified

Complementary medicine in your veterinary practice

How relevant are the following complementary medical methods for you in your veterinary practice? (What percentage of your practice is based on complementary medicine?) *

Please select one of the following answers:

Please select only one of the following answers:

- ◯ 0%
- ◯ 1-10%
- ◯ 11-20%
- ◯ 21-30%
- ◯ 31-40%
- ◯ 41-50%
- ◯ 51-60%
- ◯ 61-70%
- ◯ 71-80%
- ◯ 81-90%
- ◯ 91-100%

How do you use complementary medical methods? *

Only answer this question if the following conditions are met: Answer was '81-90%' *or* '71-80%' *or* '61-70%' *or* '51-60%' *or* '41-50%' *or* '31- 40%' *or* '21-30%' *or* '11-20%' *or* '1-10%' for question '[G04Q09]' (How relevant are complementary medicine methods for you in your veterinary work? (What percentage of your veterinary practice is accounted for by complementary medical methods?)

Please select one of the following answers:

Please select only one of the following answers:

- ◯ Complementary to conventional medicine
- ◯ I mainly use complementary medical methods
- ◯ I use complementary medicine both as a supplement and as a main focus
- ◯ Other

How often are you asked by your patient owners about complementary medicine methods? *

Please choose one of the following answers:

Please select only one of the following answers:

- ◯ Daily several times a day
- ◯ Once a day
- ◯ Several times a week
- ◯ Once a week
- ◯ Several times a month
- ◯ Once a month
- ◯ Less often
- ◯ Not specified

How many of your patient owners, in your estimation, use complementary medicine methods on their animals, either under the guidance of a veterinarian or without veterinary guidance? *

Please choose one of the following answers:

Please select only one of the following answers:

- ◯ 0-10%
- ◯ 11-20%
- ◯ 21-30%
- ◯ 31-40%
- ◯ 41-50%
- ◯ 51-60%
- ◯ 61-70%
- ◯ 71-80%
- ◯ 81-90%
- ◯ 91-100%
- ◯ Not specified

Do you think it is important that complementary medicine methods are offered in veterinary hospitals? *

Please select one of the following answers:

Please select only one of the following answers:

- 1 - not important at all
- 2
- 3
- 4
- 5
- 6
- 7
- 8
- 9
- 10 - very important
- No information

How important is the recognition of complementary medicine methods by a specialist veterinarian field/diploma from the Austrian Veterinary Chamber to you? *

Please select one of the following answers:
Please select only one of the following answers:

- 1 - not important at all
- 2
- 3
- 4
- 5
- 6
- 7
- 8
- 9
- 10 - very important
- No information

Is the number of diploma/specialist veterinary fields sufficient or should new ones be added?

Please select one of the following answers:
Please select only one of the following answers:

- Yes, is sufficient
- No, new diplomas are to be added
- No information

Which new diploma/specialist veterinary fields are to be added? *

Only answer this question if the following conditions are met:
Answer was 'no, new diplomas should be added' for question '[G01Q15]' (Is the number of diploma/veterinary specialties sufficient or should new ones be added?)

Please enter your answer here:

Are you aware of the requirement of EU Organic Regulation 2018/848 for the preferential use of phytotherapy and homeopathy among the conditions mentioned? *

Please select only one of the following answers:

- ◯ Yes
- ◯ No

How important is the integration of complementary medicine methods into the Vetmeduni curriculum to you? *

Please select one of the following answers:
Please select only one of the following answers:

- 1 - not important at all
- 2
- 3
- 4
- 5
- 6
- 7
- 8
- 9
- 10 - very important
- No information

In what form should complementary medical methods be integrated at the Vetmeduni? *

Only answer this question if the following conditions are met:
Answer was '2' *or* '3' *or* 4' *or* '5' *or* '6' *or* '7' *or* '8' *or* '9' *or* '10 - very important' for question '[G04Q18]' (How important is the integration of complementary medical methods into the curriculum of the Vetmeduni for you?)

Please select one of the following answers:
Please select only one of the following answers:

- An integral part of the degree program
- Compulsory elective subject
- Not specified
- Miscellaneous

Why is this important to you? *

Only answer this question if the following conditions are met:
Answer was 'Other' *or* 'Compulsory elective subject' *or* 'Fixed component of the degree program' for question '[G04Q19]' (In what form should complementary medical methods be integrated at the Vetmeduni?)

Please enter your answer here:

How great is the demand for complementary medicine in your practice? *

Please select one of the following answers:
Please select only one of the following answers:

- 1 - not available
- 2
- 3
- 4
- 5
- 6
- 7
- 8
- 9
- 10 - very large
- Not specified

What percentage of your patients per week do you treat with complementary medicine? *

Please select one of the following answers:

Please select only one of the following answers:

- ◯ None/only sporadically
- ◯ 1-10%
- ◯ 11-20%
- ◯ 21-30%
- ◯ 31-40%
- ◯ 41-50%
- ◯ 51-60%
- ◯ 61-70%
- ◯ 71-80%
- ◯ 81-90%
- ◯ 91-100%
- ◯ Not specified

For which illnesses do you use complementary medicine? *

Only answer this question if the following conditions are met:
Answer was '71-80%' *or* '61-70%' *or* '51-60%' *or* '41-50%' *or* '31-40%' *or* '21-30%' *or* '11-20%' *or* '1-10%' *or* '91-100%' *or* '81-90%' for question '[G04Q09]' (How relevant are complementary medicine methods for you in your veterinary practice? activity? (What percentage of your practice is based on complementary medicine?)

Please select the answers that apply:
Please select all applicable answers:

- ☐ Andrological & gynecological diseases incl. pregnancy and childbirth
- ☐ Respiratory diseases
- ☐ Dermatological diseases
- ☐ Diseases of the immune system incl. autoimmune diseases
- ☐ Feeding/housing/management-related diseases
- ☐ Gastrointestinal diseases
- ☐ Genetic diseases
- ☐ Geriatric diseases
- ☐ Hematological diseases
- ☐ Hepatological diseases
- ☐ Infectious diseases incl. parasitic diseases
- ☐ Diseases of young animals
- ☐ Cardiovascular diseases
- ☐ Neurological diseases
- ☐ Oncological diseases
- ☐ Ophthalmological diseases
- ☐ Orthopaedic diseases
- ☐ Metabolic diseases & diseases of the endocrine system
- ☐ Urological diseases
- ☐ Behavioral disorders
- ☐ Poisoning
- ☐ Injuries
- ☐ Dental diseases
- ☐ All of the diseases mentioned

☐ Other: ____________

For which of the diseases mentioned do you use complementary medicine methods most frequently? *

Only answer this question if the following conditions are met:
Answer was '91-100%' *or* '71-80%' *or* '61-70%' *or* '51-60%' *or* '41-50%' *or* '31-40%' *or* '21-30%' *or* '11-20%' *or* '1-10%' *or* '81-90%' for question '[G04Q09]' (How relevant are complementary medicine methods for you in your veterinary work? (What percentage of your veterinary practice is based on complementary medicine?)

Please choose one of the following answers:
Please select only one of the following answers:

- ◯ Andrological & gynecological diseases incl. pregnancy and childbirth
- ◯ Respiratory diseases
- ◯ Dermatological diseases
- ◯ Diseases of the immune system incl. autoimmune diseases
- ◯ Feeding/housing/management-related diseases
- ◯ Gastrointestinal diseases
- ◯ Genetic diseases
- ◯ Geriatric diseases
- ◯ Hematological diseases
- ◯ Hepatological diseases
- ◯ Infectious diseases incl. parasitic diseases
- ◯ Young animal diseases
- ◯ Cardiovascular diseases
- ◯ Neurological diseases
- ◯ Oncological diseases
- ◯ Ophthalmological diseases
- ◯ Orthopaedic diseases
- ◯ Metabolic diseases & diseases of the endocrine system
- ◯ Urological diseases
- ◯ Behavioral disorders
- ◯ Poisoning
- ◯ Injuries

- ◯ Dental diseases

Further training

How often do you take part in further training courses on complementary medicine topics or do self-study in this area? *

Please choose one of the following answers:
Please select only one of the following answers:

- ◯ Never
- ◯ Less than once a year
- ◯ Once a year
- ◯ Once per quarter
- ◯ Once per month
- ◯ Once per week
- ◯ Daily
- ◯ Not specified

How have you trained in complementary medicine methods over the past year? *

Only answer this question if the following conditions are met:
Answer was 'less than once a year' *or* 'once a year' *or* 'once a quarter' *or* 'once a month' *or* 'once a week' *or* 'daily' for question '[G05Q25]' (How often do you take part in further training courses on complementary medicine topics or do you
Self-study in this area?)

Please select the appropriate answer for each item:

	Never	Less than once a year	Once per year	Once per quarter	Once per month	Once per week	Daily
**Scientific journals**	◯	◯	◯	◯	◯	◯	◯
**Webinars**	◯	◯	◯	◯	◯	◯	◯
**Seminars**	◯	◯	◯	◯	◯	◯	◯
**Congresses**	◯	◯	◯	◯	◯	◯	◯

Should the veterinary association offer more training courses in complementary medicine? *

Please select one of the following answers:
Please select only one of the following answers:

◯ Yes
◯ No
◯ Not specified

In what form? *

Only answer this question if the following conditions are met: Answer was 'yes' to question '[G05Q27]' (Should the veterinary association offer more training courses in complementary medicine?)

Please select the answers that apply:
Please select all applicable answers:

- ☐ By training providers selected by the Chamber of Veterinarians
- ☐ Through working groups of veterinarians interested in complementary medicine
- ☐ Other: ____________

Should the training organizations offer more complementary medicine training courses? *

Please select one of the following answers:
Please select only one of the following answers:

- ◯ Yes
- ◯ No
- ◯ Not specified

Professional representation

How important are the Diploma/specialist veterinary fieldss for your own Practice/application of complementary medicine? *

Only answer this question if the following conditions are met:
Answer was '71-80%' *or* '61-70%' *or* '51-60%' *or* '41-50%' *or* '31-40%' *or* '21-30%' *or* '11-20%' *or* '1-10%' *or* '91-100%' *or* '81-90%' for question '[G04Q09]' (How relevant are complementary medicine methods for you in your veterinary practice? activity? (What percentage of your veterinary practice is based on complementary medicine?)

Please choose one of the following answers:
Please select only one of the following answers:

- ◯ 1 - not important at all
- ◯ 2
- ◯ 3
- ◯ 4
- ◯ 5
- ◯ 6
- ◯ 7
- ◯ 8
- ◯ 9
- ◯ 10 - very important

Do you consider the training provided by the respective training/professional associations for the application of the respective complementary medical method in practice to be sufficient? *

Only answer this question if the following conditions are met:
Answer was '81-90%' *or* '71-80%' *or* '61-70%' *or* '51-60%' *or* '41-50%' *or* '31-40%' *or* '21-30%' *or* '1-10%' *or* '11-20%' *or* '91-100%' for question '[G04Q09]' (How relevant are complementary medicine methods for you in your veterinary practice? activity? (What percentage of your veterinary practice is based on complementary medicine?)

Please select one of the following answers:
Please select only one of the following answers:

- ◯ 1 - No, completely inadequate
- ◯ 2
- ◯ 3
- ◯ 4
- ◯ 5
- ◯ 6
- ◯ 7
- ◯ 8
- ◯ 9
- ◯ 10 - Yes, perfectly adequate

Should complementary medicine therapies be covered by pet insurance? *

Please select one of the following answers:

Please select only one of the following answers:

- ◯ 1 - No, definitely not
- ◯ 2
- ◯ 3
- ◯ 4
- ◯ 5
- ◯ 6
- ◯ 7
- ◯ 8
- ◯ 9
- ◯ 10 – yes
- ◯ Not specified

In your opinion, how important is it to patient owners that the costs are covered so that they can take advantage of complementary medicine? *

Please select one of the following answers:
Please select only one of the following answers:

- ◯ 1 - not important at all
- ◯ 2
- ◯ 3
- ◯ 4
- ◯ 5
- ◯ 6
- ◯ 7
- ◯ 8
- ◯ 9
- ◯ 10 - very important
- ◯ No information

In your opinion, does it make sense to use complementary medical methods only under veterinary responsibility? *

Please select one of the following answers:
Please select only one of the following answers:

- ◯ Yes, absolutely
- ◯ No, non-veterinarians can also make good use of complementary medicine
- ◯ Not specified

We are happy to receive comments and suggestions! *

Please enter your answer here:

To conclude: Personal details

Gender *

Please select one of the following answers:
Please select only one of the following answers:

- Female
- Male
- Diverse

Age *

Please select one of the following answers:
Please select only one of the following answers:

- Up to 30 years
- 31-40 years
- 41-50 years
- 51-60 years
- 61-70 years
- Over 70 years

Subject-specific orientation (request for Percentage of animal species you care for in your practice). *

Please enter your answer(s) here:

Bees

Fish

Poultry

Small pets

Small animals

Horses

Pigs

Reptiles

Ruminants

Wild and zoo animals

Please enter 0 for animal species that you do not care for!

Thank you very much for your participation!

13.06.2024 - 20:22

Submission of your completed questionnaire: Thank you for answering the questionnaire.

Controller pursuant to Art. 4 Z 7 GDPR

The controllers within the meaning of the GDPR (General Data Protection Regulation) and other national data protection laws of the member states as well as other data protection regulations are

Project manager
Pia Forster
11836451@students.vetmeduni.ac.at

Prof. Dr. med. vet. Annemarie Käsbohrer
Annemarie.kaesbohrer@vetmeduni.ac.at

Dr. med. vet. Petra Weiermayer
Petra.weiermayer@vetmeduni.ac.at

Processing purpose

Collection of information as part of the above-mentioned study project.

We provide the following online questionnaire via the portal of LimeSurvey GmbH, Papenreye 63, 22453 Hamburg (Germany). By accessing the survey website, LimeSurvey receives log information, i.e. cookies that are necessary for the technical processing (intermediate storage etc.) of the survey. It is not necessary to enter your e-mail address to complete a survey at a later date!

It is also important to note that the IP addresses of participants in an online survey already considered personal data in accordance with Article 4 of the GDPR,
i.e. these may not be logged either. According to the manufacturers of LimeSurvey, this is technically suppressed from the outset in order to guarantee the data protection of the anonymous survey and a high technical security standard.

The questionnaire asks for information on gender, age, education, veterinary activity, which, even in relation to each other, do not allow us to draw any conclusions about the specific identity of the person completing the questionnaire. Your identity cannot be linked to your answers by the person responsible for the project!

We would like to remind you once again that when answering open questions, you should refrain from providing any information that could allow conclusions to be drawn about individuals, such as names.

Legal basis

Art 85, 89 in conjunction with Art 6 para 1 lit e GDPR in conjunction with § 3 Z 1, 7 & 8 Universities Act (UG) [= development of science (research and teaching); support of national and international cooperation in the field of scientific research; use and implementation of research results in practice].

Storage period

Log information is stored for the duration of the session. The data from the online survey can be archived indefinitely due to the lack of identifiability of the individual participants.

Legal information

To exercise the rights to information, deletion, etc. in accordance with Art. 15 - 20 GDPR, please contact the contact persons listed above. Due to a lack of identifiability, your rights may be restricted without the provision of further information in accordance with Art. 11 GDPR.

Please send any complaints to the Austrian data protection authority www.dsb.gv.at (http://www.dsb.gv.at).
