# Supplementary material for: CIMUVET-survey: Complementary and Integrative Medicine (CIM) use in veterinary practice in Austria and CIM education at universities in Austria, Germany and Switzerland
Source: PLoS One. 2025 Jul 2;20(7):e0327599. doi: 10.1371/journal.pone.0327599 (PMC12221077; doi:10.1371/journal.pone.0327599)
Supplement: S1B Appendix — Literature research Definition of terms ‘complementary and integrative medicine’ in human and veterinary medicine. (PDF) [file pone.0327599.s002.pdf]

Supplement 1.B.

Literature research: Definition of terms ‘complementary and integrative medicine’ in human and veterinary medicine

A comprehensive literature search with various search strategies was conducted in Pubmed in June 2024 and updated in August 2024. An additional literature search on the definition of complementary and integrative medicine specifically in the field of veterinary medicine was also carried out. The objective was to provide an overview on definitions of complementary and integrative medicine as input for a survey planned among veterinarians in Austria.

Overall, search in Pubmed with the search string ((definition) AND (complementary medicine)), yielded 4103 articles, whereas this was true for the search string ((definition) AND (integrative medicine)) for 1124 papers (Figure 1). 15 articles with reference to complementary medicine were selected for data extraction after full-text screening. Two additional articles were included in the literature analysis, as this was a scientific article on the WHO strategy for traditional medicine not listed in Pubmed and an article that was cited by already selected full texts which did not meet the search term ‘complementary medicine’, so that 17 articles were consequently selected (shown in Fig. 1). A total of seven systematic reviews, one systematic overview of reviews, one scoping review including expert opinion, one position paper, six expert opinions and one search string were counted in the literature search on complementary medicine and definition [1-17]. Three studies treated a specific indication [5, 9, 10]. Two studies dealt exclusively with definitions of terms, without including individual complementary medicine disciplines [4, 7]. Of the remaining twelve articles, acupuncture, chiropractic, physiotherapy and rehabilitation medicine, and phytotherapy were each mentioned twelve times, nutrition and dietetics and homeopathy eleven times each, and neural therapy seven times [1-3, 6, 8, 11-17].

In the literature search in Pubmed using the search string ((definition) AND (integrative medicine)), thirteen articles were identified after full-text screening and selected for data extraction. Two articles that were selected in the previous literature search regarding the definition of complementary medicine and which did not meet the search term ‘integrative medicine’ were also taken into account, as they also addressed the definition of integrative medicine, so that 15 articles were used for data extraction (shown in Fig. 1). Three systematic reviews, one systematic overview of reviews, one scoping review including expert opinion, one

position paper, eight expert opinions and one search string were counted [4-8, 10, 12-14, 16-21]. Two studies treated a specific indication [5, 10]. Four studies did not list or categorise complementary or integrative medical disciplines [4, 7, 18, 20]. Two articles did not include nutrition and dietetics in integrative medicine, although in both studies nutrition and dietetics was included in lifestyle medicine and therefore also in non-conventional therapies [6, 19]. Of the remaining nine articles, acupuncture, chiropractic, physiotherapy and rehabilitation medicine, and phytotherapy were each mentioned nine times, homeopathy eight times, nutrition and dietetics seven times and neural therapy five times [6, 8, 12-14, 16, 17, 19, 21]. Both results were presented in a table (shown in Tab. 1).

In the literature search in Pubmed, three articles were used for data extraction for the search terms ((definition) AND (complementary medicine OR integrative medicine) AND (veterinar*)) after full-text screening (shown in Fig. 2). For veterinary medicine a total of one position paper and two expert opinions were identified only [22-24]. Neither systematic nor scoping reviews were available for the definition of complementary or integrative medicine. In two of the three selected articles, including the consensus guideline for an integrative veterinary curriculum at veterinary universities in the USA, all complementary medicine methods queried in the cross-sectional study were included in the definitions of complementary and integrative medicine [23, 24]. An article that dealt with the use of integrative medicine in equine practice cited acupuncture, chiropractic, nutrition and dietetics, physiotherapy and rehabilitation medicine as examples of complementary or integrative medical treatment options [22]. Detailed results from literature search for definitions from veterinary medicine are shown in Tab. 2.

The documentation of the literature search and its outcome was performed according to the PRISMA Guideline [25].

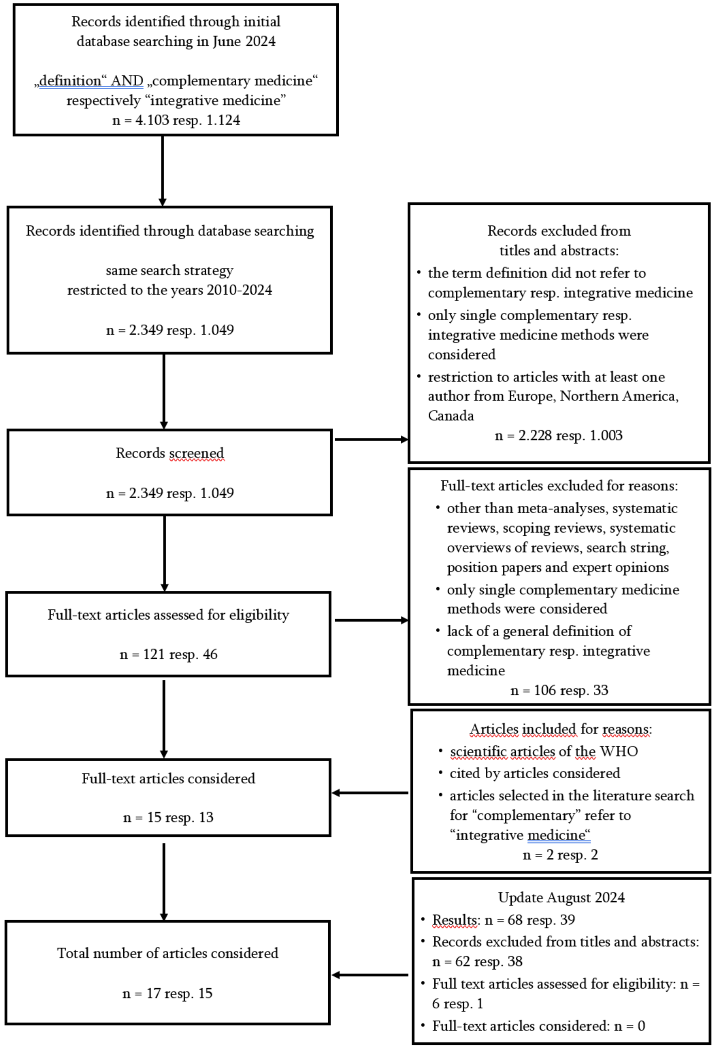

Fig. 1: Literature search "((definition) AND (complementary medicine) respectively (integrative medicine))”

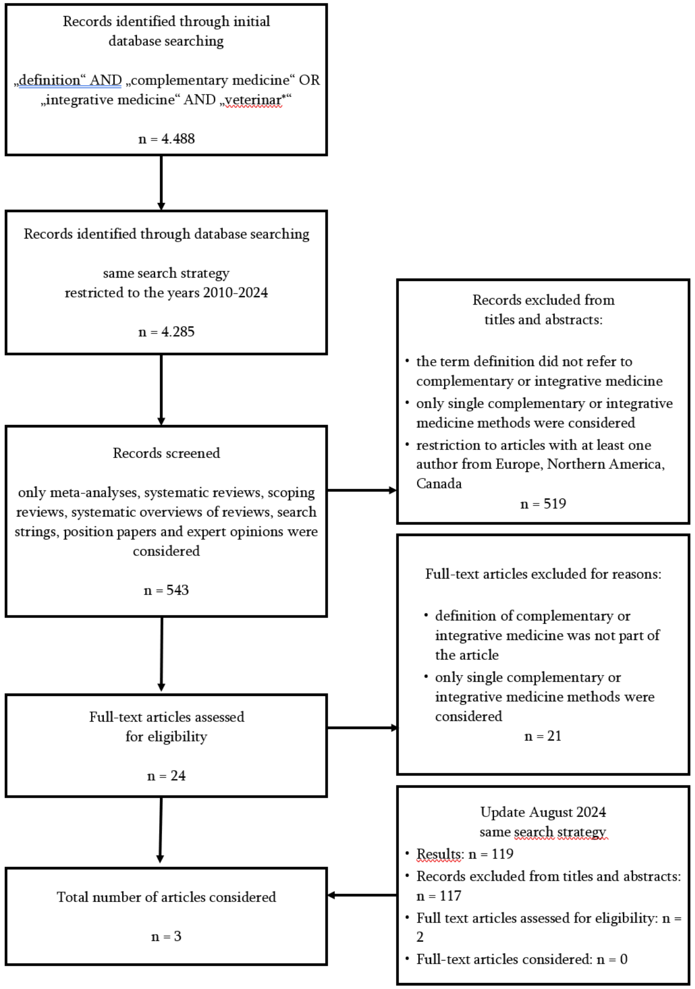

Fig. 2: Literature search ((definition) AND (complementary medicine) OR (integrative medicine) AND (veterinar*))

Tab. 1: Literature search "definition" AND "complementary medicine"

The articles were sorted by publication date in descending order (2024 to 2010). The complementary medicine disciplines included in the questionnaire study were listed in a separate column if they were included in the relevant article on complementary medicine, whereby chiropractic were counted as manual and physiotherapy and rehabilitation medicine as manual or physical medicine, since the specific terms chiropractic and physiotherapy and rehabilitation medicine were often not mentioned in the articles. Physiotherapy and rehabilitation medicine were also listed when individual sub-disciplines such as hydrotherapy, massage, etc. were mentioned. Nutrition and dietetics was also listed when dietary supplements were mentioned. In this case, the relevant complementary medicine disciplines were marked with an *. The mark ** was used when the relevant complementary medicine disciplines were mentioned in accordance with the National Center for Complementary and Integrative Health Complementary, alternative, or integrative health: What's in a name? https://www.nccih.nih.gov/health/complementary-alternative-or-integrative-health-whats-in-a-name#types. Articles were marked with *** if an evidence assessment was carried out and only those methods were listed that showed sufficient evidence for the respective clinical picture.

	Authors	Evidence class	Outcome	Complementary medicine discipline according to cross-sectional study	Reference
1.	Hoenders R., Ghelman R., Portella C., Simmons S., Locke A., Cramer H., Gallego-Perez D., Jong M.	Expert opinion	This article has discussed the status of conventional medicine, the integration of traditional and complementary medicine, the current role of integrative medicine and the remaining challenges of the World Health Organization (WHO) strategy for traditional and complementary medicine from 2014, in order to be able to contribute to the new 10-year strategy for traditional and complementary medicine of the WHO, which is scheduled to be published in 2025. In this article, a distinction is made between conventional and non-conventional medicine. Lifestyle, traditional and complementary medicine are included in non-conventional medicine. Acupuncture, mind-body medicine, anthroposophic medicine, homeopathy and phytotherapy were mentioned as examples in the context of the definition of complementary medicine. Nutrition and dietetics and	Acupuncture, chiropractic*, homeopathy, physiotherapy and rehabilitation medicine*, phytotherapy	Hoenders R, Ghelman R, Portella C, Simmons S, Locke A, Cramer H, et al.. A review of the WHO strategy on traditional, complementary, and integrative medicine from the perspective of academic consortia for integrative medicine and health. Frontiers in Medicine. 2024;11. https://www.frontiersin.org/articles/10.3389/fmed.2024.1395698/full

			exercise/physical therapy were included in lifestyle medicine. The term 'integrative medicine' was used to describe all therapeutic options that can be used safely and effectively.		
2.	Ng JY, Dhawan T, Fajardo RG, Masood HA, Sunderji S, Wieland LS, Moher D.	Expert opinion	The definitions of complementary, alternative and integrative medicine were described as dynamic, changing over time and depending on the location. The list of 604 complementary medicine methods from the article 'Operational definition of complementary, alternative and integrative medicine derived from a systematic search' was used to develop various search strings to check whether the methods mentioned also belong to complementary medicine in 2023. This operational definition was cited by Cochrane Complementary Medicine as the most up-to-date list of these therapies: https://cam.cochrane.org/operational-definition-complementary-medicine Complementary medicine methods included acupuncture, chiropractic medicine, nutrition and dietetics, homeopathy, mind-body medicine, osteopathy, physical therapy, phytotherapy, and behavioural medicine.	Acupuncture and neural therapy, chiropractic, nutrition and dietetics, homeopathy, physiotherapy and rehabilitation medicine*, phytotherapy	Ng JY, Dhawan T, Fajardo RG, Masood HA, Sunderji S, Wieland LS, et al. The Brief History of Complementary, Alternative, and Integrative Medicine Terminology and the Development and Creation of an Operational Definition. Integr Med Res. 2023;12(4):100978. https://pubmed.ncbi.nlm.nih.gov/37927333/
3.	Ng JY, Dhawan T, Dogadova E, Taghi-Zada Z, Vacca A, Fajardo RG, Masood HA, Patel R, Sunderji S, Wieland LS, Moher D.	Search string	A search string was developed based on the list of 604 complementary medicine methods from the article 'Operational definition of complementary, alternative and integrative medicine derived from a systematic search', which should enable a simpler systematic search for complementary, alternative and integrative medicine in bibliographic databases, including those of the OVID platform, the EBSCO platform, Scopus and Web of Science.	Acupuncture and neural therapy, chiropractic, nutrition and dietetics, homeopathy, physiotherapy and rehabilitation medicine*, phytotherapy	Ng JY, Dhawan T, Dogadova E, Taghi-Zada Z, Vacca A, Fajardo RG, et al. A comprehensive search string informed by an operational definition of complementary, alternative, and integrative medicine for systematic bibliographic database search strategies. BMC Complement Med Ther.

					2022;22(1):200. https://pubmed.ncbi.nlm.nih.gov/35897034/
4.	Ng JY, Dhawan T, Dogadova E, Taghi-Zada Z, Vacca A, Wieland LS, Moher D.	Systematic Review	Four types of peer-reviewed or otherwise quality-controlled sources of information were used to develop the operational definition for complementary and alternative medicine (CAM) and integrative medicine (IM): 1. peer-reviewed articles retrieved from searches of seven academic databases (MEDLINE, EMBASE, AMED, PsycINFO, CINAHL, Scopus and Web of Science); 2. the 'aims and scope' webpages of peer-reviewed CAIM journals; 3. CAIM entries in online encyclopaedias; and 4. high-ranking websites identified by searches for CAIM-related terms. Screening of eligible sources and data extraction of CAIM therapies were conducted independently and in duplicate. 604 complementary medicine methods were identified in the systematic literature search. These methods were listed in alphabetical order in a table and not assigned to categories. Complementary medicine methods included acupuncture, chiropractic, nutrition and dietetics, homeopathy, physical therapies and phytotherapy. The complete list of complementary medicine methods can be found at the following link: https://www.ncbi.nlm.nih.gov/pmc/articles/PMC9006507/table/Tab2/	Acupuncture and neural therapy, chiropractic, nutrition and dietetics, homeopathy, physiotherapy and rehabilitation medicine*, phytotherapy	Ng JY, Dhawan T, Dogadova E, Taghi-Zada Z, Vacca A, Wieland LS, et al. Operational definition of complementary, alternative, and integrative medicine derived from a systematic search. BMC Complement Med Ther. 2022;22(1):104. https://pubmed.ncbi.nlm.nih.gov/35413882/

5.	Homberg A, Scheffer C, Brinkhaus B, Fröhlich U, Huber R, Joos S, Klose P, Kramer K, Ortiz M, Rostock M, Valentini J, Stock-Schröer B.	Position paper	Complementary medicine treatment options include those methods that either prevent disease or are used to supplement conventional therapy for existing illnesses. In Germany, the term 'natural healing methods' is also used to refer to 'mind-body' therapies, nutritional medicine, hydrotherapy, phytotherapy and movement therapy, as well as neural therapy, TCM, homeopathy and anthroposophic medicine. Behavioural medicine, relaxation and stress reduction exercises, and nutrition and dietetics based on the concept of salutogenesis are also considered complementary medicine. Depending on the literature, neural therapy, acupuncture, homeopathy and anthroposophic medicine could be counted as both complementary medicine and (extended) natural healing methods.	Acupuncture and neural therapy, chiropractic, nutrition and dietetics, homeopathy, physiotherapy and rehabilitation medicine*, phytotherapy	Homberg A, Scheffer C, Brinkhaus B, Fröhlich U, Huber R, Joos S, et al. Naturopathy, complementary and integrative medicine in medical education - position paper by the GMA Committee Integrative Medicine and Perspective Pluralism. GMS J Med Educ. 2022;39(2):Doc16. https://pubmed.ncbi.nlm.nih.gov/35692361/
6.	Lee EL, Richards N, Harrison J, Barnes J.	Systematic Review	This systematic review examined national studies published between 2010 and 2019 that investigated the prevalence of traditional, complementary and alternative medicine (TCAM) use in the general population. The wide range of prevalence (24-71.3 %) in different countries is not surprising; the studies used different TCAM definitions, prevalence periods, data collection instruments and analytical approaches. TCAM is subdivided into TCAM products and TCAM therapies. TCAM products include TCAM drugs, dietary supplements and traditional Chinese medicine (TCM) drugs. TCAM therapies include, among others, 'mind-body' exercises (e.g. yoga), manual therapies (e.g. chiropractic, osteopathy), energy therapy (e.g. reiki) and methods of traditional Chinese medicine (e.g. acupuncture, Ayurvedic medicine). The surveys	Acupuncture and neural therapy, chiropractic, nutrition and dietetics, homeopathy, physiotherapy and rehabilitation medicine*, phytotherapy	Lee EL, Richards N, Harrison J, Barnes J. Prevalence of Use of Traditional, Complementary and Alternative Medicine by the General Population: A Systematic Review of National Studies Published from 2010 to 2019. Drug Saf. 2022;45(7):713-35. https://pubmed.ncbi.nlm.nih.gov/35788539/

			asked about the use of the following methods, among others: acupuncture, homeopathy, neural therapy and osteopathy.		
7.	Witt CM, Balneaves LG, Cardoso MJ, Cohen L, Greenlee H, Johnstone P, Kücük Ö, Mailman J, Mao JJ.	Scoping Review & Expert opinion	In this article, integrative oncology was defined as a patient-centered, evidence-based field of cancer care that uses mind-body therapies, natural products, and/or lifestyle changes from various traditions alongside conventional medical cancer therapies. The goal of integrative oncology has been defined as optimizing health, quality of life and clinical outcomes throughout cancer treatment and empowering people to prevent cancer and actively participate before, during and after cancer treatment.<br>It was also found that complementary medicine is often requested by patients to improve quality of life during and after cancer treatment or after cancer, to increase the effectiveness of conventional medical cancer treatment and to reduce side effects. The complementary medical therapies described in integrative oncology included acupuncture, chiropractic, the use of nutritional supplements, as well as methods to promote awareness and yoga.	Acupuncture, chiropractic, nutrition and dietetics*, physiotherapy and rehabilitation medicine**, phytotherapy	Witt CM, Balneaves LG, Cardoso MJ, Cohen L, Greenlee H, Johnstone P, et al. A Comprehensive Definition for Integrative Oncology. J Natl Cancer Inst Monogr. 2017;2017(52). https://pubmed.ncbi.nlm.nih.gov/29140493/
8.	Greenlee H, DuPont-Reyes MJ, Balneaves LG, Carlson LE, Cohen MR, Deng G, Johnson JA,	Systematic Review***	To update the previously published clinical practice guidelines for breast cancer, which were based on a systematic review of the literature from January 1, 1990, to December 31, 2013, a systematic review of published randomized controlled trials from January 1, 2014, to December 31, 2015, was conducted using the same search criteria and process. The following	Acupuncture, chiropractic*, physiotherapy and rehabilitation medicine*, phytotherapy	Greenlee H, DuPont-Reyes MJ, Balneaves LG, Carlson LE, Cohen MR, Deng G, et al. Clinical practice guidelines on the evidence-based use of integrative therapies during and after breast cancer

	Mumber M, Seely D, Zick S, Boyce L, Tripathy D.		methods with sufficient evidence were described in the context of complementary medicine treatment for breast cancer, among others: Acupuncture, acupressure, ginger, ginseng as phytotherapeutic agents, laser therapy, lymphatic drainage and massage, meditation, mistletoe extract, music therapy, reflexology, relaxation exercises, qigong, stress management and yoga. Nutrition and dietetics were explicitly not the subject of this systematic review.		treatment. CA Cancer J Clin. 2017;67(3):194-232. https://pubmed.ncbi.nlm.nih.gov/28436999/
9.	Lauche R, Cramer H, Häuser W, Dobos G, Langhorst J.	Systematic Overview of Reviews***	This systematic overview of reviews examined complementary medicine treatment options for fibromyalgia syndrome, a chronic condition. Consistently positive results were found for tai chi, yoga, meditation, hypnosis, hydrotherapy, among others. There were contradictory results for Qigong, acupuncture, chiropractic measures and dietary supplements. Inconclusive results were found for homeopathy and phytotherapy.	Physiotherapy and rehabilitation medicine	Lauche R, Cramer H, Häuser W, Dobos G, Langhorst J. A Systematic Overview of Reviews for Complementary and Alternative Therapies in the Treatment of the Fibromyalgia Syndrome. Evid Based Complement Alternat Med. 2015;2015:610615. doi: 10.1155/2015/610615Epub 2015 Jul 13. PMID: 26246841; PMCID: PMC4515506. https://pubmed.ncbi.nlm.nih.gov/26246841/
10.	Fischer F, Lewith G, Witt CM, Linde K, von Ammon K, Cardini F, Falkenberg T, Fønnebø V, Johannessen H, Reiter B,	Systematic Review	First, a systematic literature review of key topics in clinical and epidemiologic research in complementary medicine was conducted to identify the general concepts, methods, strengths and weaknesses of current complementary medicine research. These findings were discussed in a workshop with international complementary and alternative medicine (CAM) experts and strategic and methodological recommendations were defined to	Acupuncture, chiropractic, nutrition and dietetics*, homeopathy, physiotherapy and rehabilitation medicine*, phytotherapy	Fischer F, Lewith G, Witt CM, Linde K, von Ammon K, Cardini F, et al. A research roadmap for complementary and alternative medicine - what we need to know by 2020. Forsch Komplementmed. 2014;21(2):e1-16.

	Uehleke B, Weidenhammer W, Brinkhaus B.		improve the rigor and relevance of CAM research. This article could not cover the whole of Europe, but only individual member states of the European Union. The definitions of CAM varied between Member States. Consequently, differences in recognition and attitudes towards complementary medicine could also be identified. Six key research areas were proposed to be investigated in order to create a solid knowledge base and enable stakeholders to make informed decisions. These are: Research into the prevalence of CAM in Europe, research into differences in the attitudes and needs of citizens towards CAM, research into the safety of CAM, research into the comparative effectiveness of CAM, research into the effects of context and meaning, research into various models for the integration of alternative healthcare. CAM includes acupuncture, anthroposophic medicine, aromatherapy, phytotherapy, homeopathy, massage, shiatsu and yoga.		https://pubmed.ncbi.nlm.nih.gov/24851850/
11.	Eardley S, Bishop FL, Prescott P, Cardini F, Brinkhaus B, Santos-Rey K, Vas J, von Ammon K, Hegyi G, Dragan S, Uehleke B, Fønnebø V, Lewith G.	Systematic Review	A systematic literature search was conducted in Ovid MEDLINE, Cochrane Library, CINAHL, EMBASE, PsychINFO including PsychARTICLES, Web of Science, AMED and CISCOM for surveys on the use of complementary medicine. Additional studies were identified through experts and gray literature. Cross-sectional, population-based or cohort studies reporting on the use of complementary medicine in any EU language were included.<br>The most frequently described complementary medicine disciplines were: Phytotherapy (31 studies), homeopathy (25 studies), chiropractic (17 studies), acupuncture (14 studies), reflexology (11 studies), and	Acupuncture, chiropractic, nutrition and dietetics*, homeopathy, physiotherapy and rehabilitation medicine*, phytotherapy	Eardley S, Bishop FL, Prescott P, Cardini F, Brinkhaus B, Santos-Rey K, et al. A systematic literature review of complementary and alternative medicine prevalence in EU. Forsch Komplementmed. 2012;19 Suppl 2:18-28. https://pubmed.ncbi.nlm.nih.gov/23883941//

			nutritional medicine including supplements and calcium supplementation (nine studies).		
12.	Holmberg C, Brinkhaus B, Witt C.	Expert opinion	This article has established that the terms complementary and integrative medicine should be considered from four different perspectives: 1. from a medical perspective, 2. based on research, 3. from a public relations perspective and 4. from a healthcare perspective. In summary, the term "integrative medicine" refers to the combination of conventional and complementary medicine. Finally, the wish for further research into the exact definition of the term was expressed.	The individual complementary medicine methods were not the subject of the article.	Holmberg C, Brinkhaus B, Witt C. Experts' opinions on terminology for complementary and integrative medicine - a qualitative study with leading experts. BMC Complement Altern Med. 2012 Nov 14;12:218. doi: 10.1186/1472-6882-12-218. PMID: 23151006; PMCID: PMC3522550. https://pubmed.ncbi.nlm.nih.gov/23151006/
13.	Frass M, Strassl RP, Friehs H, Müllner M, Kundi M, Kaye AD.	Systematic Review	A systematic search of the available literature was conducted using PubMed/Medline, PSYNDEX, PsycLit and other databases. This systematic review revealed that the use of complementary medicine, as well as specific medical training in this area, has increased over the last decade. Acupuncture and homeopathy were among the complementary medicine disciplines used more frequently in Europe than in the USA and Canada. Overall, the following methods were used most frequently: chiropractic, homeopathy, phytotherapy and massage. It was also found that the demand for complementary medicine and the use of the individual methods have increased.	Acupuncture and neural therapy, chiropractic, nutrition and dietetics, homeopathy, physiotherapy and rehabilitation medicine*, phytotherapy	Frass M, Strassl RP, Friehs H, Müllner M, Kundi M, Kaye AD. Use and acceptance of complementary and alternative medicine among the general population and medical personnel: a systematic review. Ochsner J. 2012 Spring;12(1):45-56. PMID: 22438782; PMCID: PMC3307506. https://pubmed.ncbi.nlm.nih.gov/22438782/

14.	Gaboury I, April KT, Verhoef M.	Expertenmeinung	Complementary medicine was defined as a wide range of treatment options (e.g. chiropractic and osteopathy) developed and carried out by medical professionals. Three points were defined for the aim of complementary medical treatment: 1. the whole person is the focus of treatment, 2. the person can promote health individually and 3. the body has the opportunity to heal itself. Integrative healthcare was defined as a system that is patient-centered and interdisciplinary (encompassing both conventional and complementary medicine disciplines that are safe, effective and evidence-based) to facilitate optimal health and healing.	The individual complementary medicine methods were not the subject of the article.	Gaboury I, April KT, Verhoef M. A qualitative study on the term CAM: is there a need to reinvent the wheel? BMC Complement Altern Med. 2012 Aug 21;12:131. doi: 10.1186/1472-6882-12-131. PMID: 22909051; PMCID: PMC3462712. https://pubmed.ncbi.nlm.nih.gov/22909051/
15.	Falkenberg T, Lewith G, Roberti di Sarsina P, von Ammon K, Santos-Rey K, Hök J, Frei-Erb M, Vas J, Saller R, Uehleke B.	Expertenmeinung	The working group consisted of active researchers in the field of complementary and alternative medicine from six European countries. A simplified version of the consensus-based decision-making model was used in the process of reaching a definition. Rough consensus was sought to maximize the likelihood that the views of all group members would be considered. PubMed was systematically searched for definitions of CAM produced by various stakeholders, including citizens, patients and providers, as well as global, European and national government agencies and academic institutions. The following search terms were used without language restriction: “definition”, “terminology” AND “CAM”. Based on the various CAM definitions found and their historical, cultural and geographical backgrounds, the proposed definition was developed from several rounds of discussion at a final project consensus meeting in May 2012. The World Health Organization (WHO) definition from the year 2000 was selected as the most important basis	Acupuncture and neural therapy, chiropractic, nutrition and dietetics, homeopathy, physiotherapy and rehabilitation medicine*, phytotherapy	Falkenberg T, Lewith G, Roberti di Sarsina P, et al. Towards a pan-European definition of complementary and alternative medicine--a realistic ambition?. Forsch Komplementmed. 2012;19 Suppl 2:6-8. doi:10.1159/000343812 https://pubmed.ncbi.nlm.nih.gov/23883939/

			for the development of a pan-European definition, as it is relevant worldwide and is supported by the WHO. The following pan-European definition was created: Complementary and alternative medicine (CAM) used by European citizens encompasses a variety of different medical systems and therapies based on knowledge, skills and practices derived from theories, philosophies and experiences and used to maintain and improve health and to prevent, diagnose, alleviate or treat physical and mental illness. It has been established that CAM is mainly used outside the conventional health care system, but in some countries certain treatments are adopted or adapted by conventional healthcare. The definition also takes into account the unique European tradition of medicine with its ancient Greek and Roman "humoral" roots, including herbal medicine, manual methods, exercise and healthy eating.<br>The proposed definition does not differentiate between the origin of a CAM therapy used or whether it is offered by medical doctors or alternative practitioners and includes all CAM methods used in Europe.		
16.	Wieland LS, Manheimer E, Berman BM.	Expert opinion	In this study, with the aim of developing and classifying an operational definition of complementary and alternative medicine for the Cochrane Collaboration, complementary medicine methods were assigned to the five categories of the National Center for Complementary and Alternative Medicine (NCAAM): "mind-body" medicine, therapies based on natural products, manual therapies, energy treatments and holistic medicine. For example, the following therapies were assigned to the	Acupuncture, chiropractic, nutrition and dietetics, homeopathy, physiotherapy and rehabilitation medicine*, phytotherapy	Wieland LS, Manheimer E, Berman BM. Development and classification of an operational definition of complementary and alternative medicine for the Cochrane collaboration. Altern Ther Health Med. 2011 Mar-Apr;17(2):50-9. PMID: 21717826; PMCID:

			complementary medicine categories: Acupuncture, nutritional medicine, homeopathy and osteopathy.		PMC3196853. https://pubmed.ncbi.nlm.nih.gov/21717826/
17.	Jeppesen E, Juvet LK.	Systematic Review***	This article comprises a systematic literature search in February 2010 for systematic reviews on cancer and alternative medicine from the following databases: The Cochrane Library, DARE, Science Citation Index, Medline, EMBASE, Pedro, Amed and PsycINFO. Two individuals independently read all unique titles and abstracts identified in the literature search and assessed relevance in relation to the inclusion and exclusion criteria. The methodological quality of the studies was assessed independently by two review authors. The quality of evidence for the main outcomes was graded according to GRADE where appropriate. It was not possible to summarize the efficacy and safety results for all selected primary outcomes or measures. Articles were categorized according to the different complementary medicine therapies. The literature search for systematic reviews identified 2199 titles, of which 39 met the inclusion criteria. These systematic reviews assessed the efficacy or safety of complementary and/or alternative medicine therapies used in cancer patients. Most studies examined complementary medicine therapies in addition to standard therapy. For example, acupuncture, massage, nutritional supplements and phytotherapy were discussed. In general, the quality of the evidence was low.	Phytotherapy	Jeppesen E, Juvet LK. Complementary and Alternative Medicine for Patients with Cancer [Internet]. Oslo, Norway: Knowledge Centre for the Health Services at The Norwegian Institute of Public Health (NIPH); 2011 Jun. Report from Norwegian Knowledge Centre for the Health Services (NOKC) No. 12-2011. PMID: 29320042. https://pubmed.ncbi.nlm.nih.gov/29320042/

Tab. 2: Literature search "definition" AND "integrative medicine"

The articles were sorted by publication date in descending order (2024 to 2010). The complementary medicine disciplines included in the questionnaire study were listed in a separate column if they were included in the relevant article on complementary medicine, whereby chiropractic were counted as manual and physiotherapy and rehabilitation medicine as manual or physical medicine, since the specific terms chiropractic and physiotherapy and rehabilitation medicine were often not mentioned in the articles. Physiotherapy and rehabilitation medicine were also listed when individual sub-disciplines such as hydrotherapy, massage, etc. were mentioned. Nutrition and dietetics were also listed when dietary supplements were mentioned. In this case, the relevant complementary medicine disciplines were marked with an *. The mark ** was used when the relevant complementary medicine disciplines were mentioned in accordance with the National Center for Complementary and Integrative Health Complementary, alternative, or integrative health: What's in a name? https://www.nccih.nih.gov/health/complementary-alternative-or-integrative-health-whats-in-a-name#types. Articles were marked with *** if an evidence assessment was carried out and only those methods were listed that showed sufficient evidence for the respective clinical picture.

	Authors	Evidence class	Outcome	Integrative medicine discipline according to cross-sectional study	Reference
1.	Hoenders R., Ghelman R., Portella C., Simmons S., Locke A., Cramer H., Gallego-Perez D., Jong M.	Expert opinion	See above	Acupuncture, chiropractic*, homeopathy, physiotherapy and rehabilitation medicine*, phytotherapy	Hoenders R, Ghelman R, Portella C, Simmons S, Locke A, Cramer H, et al.. A review of the WHO strategy on traditional, complementary, and integrative medicine from the perspective of academic consortia for integrative medicine and health. Frontiers in Medicine. 2024;11. https://www.frontiersin.org/a

					rticles/10.3389/fmed.2024.1395698/full
2.	Hunter J, Harnett JE, Chan WJ, Pirotta M.	Expert opinion	This article focuses on the work of a multidisciplinary team that used an iterative approach, informed by expert knowledge and literature searches, to establish decision criteria for categorising terms in the Australian clinical interface terminology, the International Classification of Primary Care, second edition (ICPC-2 PLUS) and the Coding Atlas of Pharmaceutical Substances, depending on whether they reflect integrative medicine (IM), conventional/mainstream medicine (MM) or both IM and MM (IM/MM).<br>The iterative process included the following: (1) familiarisation with the two coding systems and their structures, (2) application of the research team's expert knowledge, supplemented by the literature, to formulate a preliminary criterion for selecting the relevant ICPC-2-PLUS-nursing process terms and CAPS terms, (3) conducting preliminary screenings of the two coding systems, (4) critically evaluating and refining the criteria, and (5) finally determining the decision criteria.<br>On the basis of the decision criteria to be used for categorising the nursing process terms in the ICPC-2 PLUS coding system, a tabular overview of the term definitions ‘integrative medicine’, ‘mainstream	Acupuncture, chiropractic, homeopathy, physiotherapy and rehabilitation medicine*, phytotherapy	Hunter J, Harnett JE, Chan WJ, Pirotta M. What is integrative medicine? Establishing the decision criteria for an operational definition of integrative medicine for general practice health services research in Australia. Integr Med Res. 2023;12(4):100995. https://pubmed.ncbi.nlm.nih.gov/37915439/

			medicine' and the combination of integrative and mainstream medicine was created. https://www.ncbi.nlm.nih.gov/pmc/articles/PMC10616154/table/tbl0002/ Integrative medicine includes, among other things, methods of traditional medicine (e.g. acupuncture), naturopathy, phytotherapy, homeopathy, manual therapies (osteopathy, chiropractic and craniosacral therapy), 'mind-body' therapies (including yoga, relaxation, meditation, music therapy, reiki kinesiology). Diet, exercise, stress and weight management, sleep and environment were cited as examples of lifestyle medicine and of the combined use of integrative and 'mainstream' medicine.		
3.	Ng JY, Dhawan T, Fajardo RG, Masood HA, Sunderji S, Wieland LS, Moher D.	Expert opinion	See above	Acupuncture and neural therapy, chiropractic, nutrition and dietetics, homeopathy, physiotherapy and rehabilitation medicine*, phytotherapy	Ng JY, Dhawan T, Fajardo RG, Masood HA, Sunderji S, Wieland LS, et al. The Brief History of Complementary, Alternative, and Integrative Medicine Terminology and the Development and Creation of an Operational Definition. Integr Med Res. 2023;12(4):100978. https://pubmed.ncbi.nlm.nih.gov/37927333/
4.	Ng JY, Dhawan T, Dogadova E, Taghi-Zada Z, Vacca A, Fajardo RG, Masood HA,	Search string	See above	Acupuncture and neural therapy, chiropractic, nutrition and dietetics, homeopathy,	Ng JY, Dhawan T, Dogadova E, Taghi-Zada Z, Vacca A, Fajardo RG, et al. A comprehensive search string informed by an operational definition of complementary, alternative,

	Patel R, Sunderji S, Wieland LS, Moher D.			physiotherapy and rehabilitation medicine*, phytotherapy	and integrative medicine for systematic bibliographic database search strategies. BMC Complement Med Ther. 2022;22(1):200. https://pubmed.ncbi.nlm.nih.gov/35897034/
5.	Ng JY, Dhawan T, Dogadova E, Taghi-Zada Z, Vacca A, Wieland LS, Moher D.	Systematic Review	See above	Acupuncture and neural therapy, chiropractic, nutrition and dietetics, homeopathy, physiotherapy and rehabilitation medicine*, phytotherapy	Ng JY, Dhawan T, Dogadova E, Taghi-Zada Z, Vacca A, Wieland LS, et al. Operational definition of complementary, alternative, and integrative medicine derived from a systematic search. BMC Complement Med Ther. 2022;22(1):104. https://pubmed.ncbi.nlm.nih.gov/35413882/
6.	Homberg A, Scheffer C, Brinkhaus B, Fröhlich U, Huber R, Joos S, Klose P, Kramer K, Ortiz M, Rostock M, Valentini J, Stock-Schröer B.	Position paper	See above	Acupuncture and neural therapy, chiropractic, nutrition and dietetics, homeopathy, physiotherapy and rehabilitation medicine*, phytotherapy	Homberg A, Scheffer C, Brinkhaus B, Fröhlich U, Huber R, Joos S, et al. Naturopathy, complementary and integrative medicine in medical education - position paper by the GMA Committee Integrative Medicine and Perspective Pluralism. GMS J Med Educ. 2022;39(2):Doc16. https://pubmed.ncbi.nlm.nih.gov/35692361/

7.	Kienle GS, Ben-Arye E, Berger B, Cuadrado Nahum C, Falkenberg T, Kapócs G, Kiene H, Martin D, Wolf U, Szöke H.	Expert opinion	A three-step consensus process was used to develop the research strategy, based on the Guidance for Developers of Health Research Reporting Guidelines. This consisted of (1) pre-meeting literature searches, firstly to inform the recommendation for whole-system health research, and secondly to analyse what goals have been studied to what extent in anthroposophic medicine (AM); this was followed by interviews with key stakeholders to strategy or revise it, add rationale and supporting references, and tailor the strategy to AM research; (2) face-to-face consensus meetings to further develop and refine the strategy; and (3) post-meeting feedback followed by finalisation. According to the definition described in this article, traditional and complementary medicine (T&CM) is used for both health maintenance and disease prevention as well as for chronic, non-communicable diseases. The term 'integrative medicine' (IM) encompasses the combination of the disciplines of mind-body, traditional and complementary medicine with conventional therapy options. IM systems are characterised, among other things, by the following features: an emphasis on salutogenesis, the 'natural healing power' of the organism; a holistic understanding of the human being; an emphasis on lifestyle changes; extensive use of non-pharmacological interventions; a strong emphasis on the therapeutic relationship between doctor and patient; joint clinical decision-making based on evidence; use of both conventional and complementary medical treatments. A research strategy that does justice to this complexity should	The individual complementary and integrative medical methods were not the subject of the article.	Kienle GS, Ben-Arye E, Berger B, et al. Contributing to Global Health: Development of a Consensus-Based Whole Systems Research Strategy for Anthroposophic Medicine. Evid Based Complement Alternat Med. 2019;2019:3706143. Published 2019 Nov 12. doi:10.1155/2019/3706143 https://pubmed.ncbi.nlm.nih.gov/31781267/

			include elements focusing on (I) efficacy and effectiveness, subdivided into (a) the evaluation of the multimodal and multidisciplinary medical system as a whole or of a complex multimodal therapy concept, (b) an appropriate number of methodologically rigorous, confirmatory randomised controlled studies on exemplary pharmacological and non-pharmacological therapies and indications, (c) a broad spectrum of interventions and patient-centred care strategies with less extensive formats such as well-conducted small studies, observational studies and high-quality case reports and series or subgroup analyses from overall system studies or health services research; (II) Safety; (III) Economics; (IV) Evidence synthesis; (V) Methodological issues; (VI) Biomedical, physiological, pharmacological, pharmaceutical, psychological, anthropological and nosological issues, as well as innovation and development; (VI) patient perspective and participation, public needs and ethics; (VII) educational issues and professionalism; and (IX) disease prevention, health promotion and public health. Examples of T&CM methods that have gained significant attention in medicine include acupuncture, mind-body medicine techniques (e.g. meditation and yoga), and the use of natural products. The T&CM methods have been widely studied, developed, tested and verified using scientific methods supported by research networks (e.g. WHO, CAMbrella). The Cochrane Library lists more than 460 Cochrane reviews and more than 26,000 randomised controlled trials on T&CM.		

8.	Madsen C, Vaughan M, Koehlmoos TP.	Expert opinion	This article describes integrative medicine (IM) as a current health paradigm that promotes a ‘holistic’ approach to health through the coordinated use of appropriate therapies both within and outside of conventional medicine. The American Board of Physician Specialties describes IM in terms of five domains: partnership between patient and physician; consideration of all factors, including mental, physical, and spiritual, in the management of health, well-being, and disease; promotion of the body's own healing response through the use of conventional and non-conventional methods; use of less invasive and less harmful treatments whenever possible to treat the patient as a whole and not just the disease; and the ideal of scientifically based, research-oriented medicine that is willing to critically examine new paradigms.<br>The National Center for Complementary and Integrative Health (NCCIH) at the National Institutes of Health (NIH) defines nonconventional treatments as alternative when used in place of usual therapies, complementary when used in addition to usual therapies, and integrative when used as part of a coordinated system of care.<br>The article is based on three surveys conducted in 2005 and 2009, and 2013 and 2010–2015 on the use of integrative medicine in the military health system (MHS). These surveys identified 23 different integrative medicine methods in the MHS. These included acupuncture, chiropractic medicine, osteopathy, hypnosis, massage, meditation, naturopathy, nutritional counselling, behavioural therapy and yoga.	Acupuncture, chiropractic, nutrition and dietetics, homeopathy, physiotherapy and rehabilitation medicine*, phytotherapy	Madsen C, Vaughan M, Koehlmoos TP. Use of Integrative Medicine in the United States Military Health System. Evid Based Complement Alternat Med. 2017;2017:9529257. doi: 10.1155/2017/9529257. Epub 2017 Jun 13. PMID: 28690665; PMCID: PMC5485330. https://pubmed.ncbi.nlm.nih.gov/28690665/

9.	Witt CM, Balneaves LG, Cardoso MJ, Cohen L, Greenlee H, Johnstone P, Kücük Ö, Mailman J, Mao JJ.	Scoping Review & Experten opinion	See above	Acupuncture, chiropractic, nutrition and dietetics, physiotherapy and rehabilitation medicine*, phytotherapy	Witt CM, Balneaves LG, Cardoso MJ, Cohen L, Greenlee H, Johnstone P, et al. A Comprehensive Definition for Integrative Oncology. J Natl Cancer Inst Monogr. 2017;2017(52). https://pubmed.ncbi.nlm.nih.gov/29140493/
10.	Greenlee H, DuPont-Reyes MJ, Balneaves LG, Carlson LE, Cohen MR, Deng G, Johnson JA, Mumber M, Seely D, Zick S, Boyce L, Tripathy D.	Systematic Review***	See above	Acupuncture, chiropractic*, physiotherapy and rehabilitation medicine*, phytotherapy	Greenlee H, DuPont-Reyes MJ, Balneaves LG, Carlson LE, Cohen MR, Deng G, et al. Clinical practice guidelines on the evidence-based use of integrative therapies during and after breast cancer treatment. CA Cancer J Clin. 2017;67(3):194-232. https://pubmed.ncbi.nlm.nih.gov/28436999/
11.	Lauche R, Cramer H, Häuser W, Dobos G, Langhorst J.	Systematic Overview of Reviews***	See above	Physiotherapy and rehabilitation medicine	Lauche R, Cramer H, Häuser W, Dobos G, Langhorst J. A Systematic Overview of Reviews for Complementary and Alternative Therapies in the Treatment of the Fibromyalgia Syndrome. Evid Based Complement Alternat Med. 2015;2015:610615. doi: 10.1155/2015/610615. Epub 2015 Jul 13. PMID: 26246841; PMCID: PMC4515506.

					https://pubmed.ncbi.nlm.nih.gov/26246841/
12.	Coulter ID, Khorsan R, Crawford C, Hsiao AF.	Systematisc Review	The term ‘complementary medicine’ was added to the list of ‘Medical Subject Headings’ (MeSH) in 1986 and redefined by the ‘National Library of Medicine’ in 2002. According to MeSH, complementary medicine includes those methods that are not currently considered an integral part of conventional allopathic medical practice. The better the methods are researched, the more they are accepted; this applies, for example, to physical therapies, dietetics and acupuncture. The MeSH topics include acupuncture, holistic care and manipulation of the musculoskeletal system. The term ‘integrative medicine’ was introduced to MeSH in 2009 and defined as: discipline that deals with combining complementary and conventional medicine to treat the biological, psychological, social, and spiritual aspects of health and disease.<br>PubMed, Allied and Complementary Medicine (AMED), BIOSIS Previews, EMBASE, the entire Cochrane Library, MANTIS, Social SciSearch, SciSearch Cited Ref Sci, PsychInfo, CINAHL and NCCAM Grantee Publication Directories were searched from the beginning of the database until May 2009 and available studies published in English were considered. The systematic search includes the search terms ‘integrat*’ and ‘medicine’; ‘integrat*’	The individual complementary and integrative medicine methods were not the subject of the article.	Coulter ID, Khorsan R, Crawford C, Hsiao AF. Challenges of systematic reviewing integrative health care. Integr Med Insights. 2013 Jun 27;8:19-28. doi: 10.4137/IMI.S11570. PMID: 23843689; PMCID: PMC3700987. https://pubmed.ncbi.nlm.nih.gov/23843689/

			and ‘health*’ (for healthcare provision); ‘multidisciplinary care’; ‘complementary or alternative and conventional medicine or health care’ and ‘delivery of health care and integrat*’. In 2011, a literature search was conducted in PubMed for the term ‘integrative medicine’, which yielded 5235 results, of which 246 articles described clinical studies.		
13.	Holmberg C, Brinkhaus B, Witt C.	Expert opinion	See above	The individual complementary and integrative medicine methods were not the subject of the article.	Holmberg C, Brinkhaus B, Witt C. Experts' opinions on terminology for complementary and integrative medicine - a qualitative study with leading experts. BMC Complement Altern Med. 2012 Nov 14;12:218. doi: 10.1186/1472-6882-12-218. PMID: 23151006; PMCID: PMC3522550. https://pubmed.ncbi.nlm.nih.gov/23151006/
14.	Gaboury I, April KT, Verhoef M.	Expert opinion	See above	The individual complementary and integrative medicine methods were not the subject of the article.	Gaboury I, April KT, Verhoef M. A qualitative study on the term CAM: is there a need to reinvent the wheel? BMC Complement Altern Med. 2012 Aug 21;12:131. doi: 10.1186/1472-6882-12-131. PMID: 22909051; PMCID: PMC3462712. https://pubmed.ncbi.nlm.nih.gov/22909051/

15.	Falkenberg T, Lewith G, Roberti di Sarsina P, von Ammon K, Santos-Rey K, Hök J, Frei-Erb M, Vas J, Saller R, Uehleke B.	Expert opinion	See above	Acupuncture and neural therapy, chiropractic, nutrition and dietetics, homeopathy, physiotherapy and rehabilitation medicine*, phytotherapy	Falkenberg T, Lewith G, Roberti di Sarsina P, et al. Towards a pan-European definition of complementary and alternative medicine--a realistic ambition?. Forsch Komplementmed. 2012;19 Suppl 2:6-8. doi:10.1159/000343812 https://pubmed.ncbi.nlm.nih.gov/23883939/

Tab. 3: Complementary medicine results at a glance

The articles were sorted by publication date in descending order (2024 to 2010). The complementary medicine disciplines included in the questionnaire study were listed in a separate column if they were included in the relevant article on complementary medicine, whereby chiropractic were counted as manual and physiotherapy and rehabilitation medicine as manual or physical medicine, since the specific terms chiropractic and physiotherapy and rehabilitation medicine were often not mentioned in the articles. Physiotherapy and rehabilitation medicine were also listed when individual sub-disciplines such as hydrotherapy, massage, etc. were mentioned. Nutrition and dietetics were also listed when dietary supplements were mentioned. In this case, the relevant complementary medicine disciplines were marked with an *. The mark ** was used when the relevant complementary medicine disciplines were mentioned in accordance with the National Center for Complementary and Integrative Health Complementary, alternative, or integrative health: What's in a name? https://www.nccih.nih.gov/health/complementary-alternative-or-integrative-health-whats-in-a-name#types. Articles were marked with *** if an evidence assessment was carried out and only those methods were listed that showed sufficient evidence for the respective clinical picture.

Reference	Acupuncture	Chiropractic*	Homeopathy	Neural therapy	Nutrition and dietetics	Physiotherapy and rehabilitation medicine*	Phytotherapy
1. Hoenders et al., 2024	x	x	x	-	-	x	x
2. Ng et al., 2023	x	x	x	x	x	x	x
3. Ng et al., 2022a	x	x	x	x	x	x	x
4. Ng et al., 2022b	x	x	x	x	x	x	x
5. Homberg et al., 2022	x	x	x	x	x	x	x
6. Lee et al., 2019	x	x	x	x	x	x	x
7. Witt et al., 2017	x	x	-	-	x	x	x
8. Greenlee et al., 2017***	(x)	(x)	-	-	-	(x)	(x)
9. Lauche et al., 2015***	-	-	-	-	-	(x)	-
10. Fischer et al., 2014	x	x	x	-	x	x	x
11. Eardley et al., 2012	x	x	x	-	x	x	x
12. Holmberg et al., 2012**	-	-	-	-	-	-	-
13. Frass et al., 2012	x	x	x	x	x	x	x
14. Gaboury et al., 2012**	-	-	-	-	-	-	-
15. Falkenberg et al., 2012	x	x	x	x	x	x	x
16. Wieland et al., 2011	x	x	x	-	x	x	x
17. Jeppesen & Juvet, 2011***	-	-	-	-	-	-	(x)
	12	12	11	7	11	12	12

Tab. 4: Integrative medicine results at a glance

The articles were sorted by publication date in descending order (2024 to 2010). The complementary medicine disciplines included in the questionnaire study

were listed in a separate column if they were included in the relevant article on complementary medicine, whereby chiropractic were counted as manual and physiotherapy and rehabilitation medicine as manual or physical medicine, since the specific terms chiropractic and physiotherapy and rehabilitation medicine were often not mentioned in the articles. Physiotherapy and rehabilitation medicine were also listed when individual sub-disciplines such as hydrotherapy, massage, etc. were mentioned. Nutrition and dietetics were also listed when dietary supplements were mentioned. In this case, the relevant complementary medicine disciplines were marked with an *. The mark ** was used when the relevant complementary medicine disciplines were mentioned in accordance with the National Center for Complementary and Integrative Health Complementary, alternative, or integrative health: What's in a name? https://www.nccih.nih.gov/health/complementary-alternative-or-integrative-health-whats-in-a-name#types. Articles were marked with *** if an evidence assessment was carried out and only those methods were listed that showed sufficient evidence for the respective clinical picture.

Reference	Acupuncture	Chiropractic*	Homeopathy	Neural therapy	Nutrition and dietetics	Physiotherapy and rehabilitation medicine*	Phytotherapy
1. Hoenders et al., 2024	x	x	x	-	-	x	x
2. Hunter et al., 2023	x	x	x	-	-	x	x
3. Ng et al., 2023	x	x	x	x	x	x	x
4. Ng et al., 2022a	x	x	x	x	x	x	x
5. Ng et al., 2022b	x	x	x	x	x	x	x
6. Homberg et al., 2022	x	x	x	x	x	x	x
7. Kienle et al., 2019**	-	-	-	-	-	-	-
8. Madsen et al., 2017	x	x	x	-	x	x	x
9. Witt et al., 2017	x	x	-	-	x	x	x
10. Greenlee et al., 2017***	(x)	(x)	-	-	-	(x)	(x)
11. Lauche et al., 2015***	-	-	-	-	-	(x)	-
12. Coulter et al., 2013**	-	-	-	-	-	-	-
13. Holmberg et al., 2012**	-	-	-	-	-	-	-
14. Gaboury et al., 2012**	-	-	-	-	-	-	-
15. Falkenberg et al., 2012	x	x	x	x	x	x	x

	9	9	8	5	7	9	9

References

1. Eardley S, Bishop FL, Prescott P, Cardini F, Brinkhaus B, Santos-Rey K, et al. A systematic literature review of complementary and alternative medicine prevalence in EU. Forsch Komplementmed. 2012;19 Suppl 2:18-28. doi: 10.1159/000342708
2. Fischer F, Lewith G, Witt CM, Linde K, von Ammon K, Cardini F, et al. A research roadmap for complementary and alternative medicine - what we need to know by 2020. Forsch Komplementmed. 2014;21(2):e1-16. doi: 10.1159/000360744
3. Frass M, Strassl RP, Friehs H, Müllner M, Kundi M, Kaye AD. Use and acceptance of complementary and alternative medicine among the general population and medical personnel: a systematic review. Ochsner J. 2012;12(1):45-56. doi:
4. Gaboury I, April KT, Verhoef M. A qualitative study on the term CAM: is there a need to reinvent the wheel? BMC Complement Altern Med. 2012;12:131. doi: 10.1186/1472-6882-12-131
5. Greenlee H, DuPont-Reyes MJ, Balneaves LG, Carlson LE, Cohen MR, Deng G, et al. Clinical practice guidelines on the evidence-based use of integrative therapies during and after breast cancer treatment. CA Cancer J Clin. 2017;67(3):194-232. doi: 10.3322/caac.21397

6. Hoenders R, Ghelman R, Portella C, Simmons S, Locke A, Cramer H, et al. A review of the WHO strategy on traditional, complementary, and integrative medicine from the perspective of academic consortia for integrative medicine and health. Frontiers in Medicine. 2024;11. doi: 10.3389/fmed.2024.1395698
7. Holmberg C, Brinkhaus B, Witt C. Experts' opinions on terminology for complementary and integrative medicine - a qualitative study with leading experts. BMC Complement Altern Med. 2012;12:218. doi: 10.1186/1472-6882-12-218
8. Homberg A, Scheffer C, Brinkhaus B, Fröhlich U, Huber R, Joos S, et al. Naturopathy, complementary and integrative medicine in medical education - position paper by the GMA Committee Integrative Medicine and Perspective Pluralism. GMS J Med Educ. 2022;39(2):Doc16. doi: 10.3205/zma001537
9. Jeppesen E, Juvet LK. NIPH Systematic Reviews: Executive Summaries. Complementary and Alternative Medicine for Patients with Cancer. Oslo, Norway: Knowledge Centre for the Health Services at The Norwegian Institute of Public Health (NIPH)
Copyright ©2011 by The Norwegian Institute of Public Health (NIPH). 2011.
10. Lauche R, Cramer H, Häuser W, Dobos G, Langhorst J. A Systematic Overview of Reviews for Complementary and Alternative Therapies in the Treatment of the Fibromyalgia Syndrome. Evid Based Complement Alternat Med. 2015;2015:610615. doi: 10.1155/2015/610615
11. Lee EL, Richards N, Harrison J, Barnes J. Prevalence of Use of Traditional, Complementary and Alternative Medicine by the General Population: A Systematic Review of National Studies Published from 2010 to 2019. Drug Saf. 2022;45(7):713-35. doi: 10.1007/s40264-022-01189-w
12. Ng JY, Dhawan T, Dogadova E, Taghi-Zada Z, Vacca A, Fajardo RG, et al. A comprehensive search string informed by an operational definition of complementary, alternative, and integrative medicine for systematic bibliographic database search strategies. BMC Complement Med Ther. 2022;22(1):200. doi: 10.1186/s12906-022-03683-1
13. Ng JY, Dhawan T, Dogadova E, Taghi-Zada Z, Vacca A, Wieland LS, et al. Operational definition of complementary, alternative, and integrative medicine derived from a systematic search. BMC Complement Med Ther. 2022;22(1):104. doi: 10.1186/s12906-022-03556-7
14. Ng JY, Dhawan T, Fajardo RG, Masood HA, Sunderji S, Wieland LS, et al. The Brief History of Complementary, Alternative, and Integrative Medicine Terminology and the Development and Creation of an Operational Definition. Integr Med Res. 2023;12(4):100978. doi: 10.1016/j.imr.2023.100978
15. Wieland LS, Manheimer E, Berman BM. Development and classification of an operational definition of complementary and alternative medicine for the Cochrane collaboration. Altern Ther Health Med. 2011;17(2):50-9. doi:
16. Witt CM, Balneaves LG, Cardoso MJ, Cohen L, Greenlee H, Johnstone P, et al. A Comprehensive Definition for Integrative Oncology. J Natl Cancer Inst Monogr. 2017;2017(52). doi: 10.1093/jncimonographs/lgx012
17. Falkenberg T, Lewith G, Roberti di Sarsina P, von Ammon K, Santos-Rey K, Hök J, et al. Towards a pan-European definition of complementary and alternative medicine--a realistic ambition? Forsch Komplementmed. 2012;19 Suppl 2:6-8. doi: 10.1159/000343812
18. Coulter ID, Khorsan R, Crawford C, Hsiao AF. Challenges of systematic reviewing integrative health care. Integr Med Insights. 2013;8:19-28. doi: 10.4137/imi.S11570
19. Hunter J, Harnett JE, Chan WJ, Pirotta M. What is integrative medicine? Establishing the decision criteria for an operational definition of integrative medicine for general practice health services research in Australia. Integr Med Res. 2023;12(4):100995. doi: 10.1016/j.imr.2023.100995
20. Kienle GS, Ben-Arye E, Berger B, Cuadrado Nahum C, Falkenberg T, Kapócs G, et al. Contributing to Global Health: Development of a Consensus-Based Whole Systems Research Strategy for Anthroposophic Medicine. Evid Based Complement Alternat Med. 2019;2019:3706143. doi: 10.1155/2019/3706143

21. Madsen C, Vaughan M, Koehlmoos TP. Use of Integrative Medicine in the United States Military Health System. Evid Based Complement Alternat Med. 2017;2017:9529257. doi: 10.1155/2017/9529257
22. Haussler KK. Integrative Medicine in Equine Practice. Vet Clin North Am Equine Pract. 2022;38(3):445-53. doi: 10.1016/j.cveq.2022.06.003
23. Memon MA, Shmalberg J, Adair HS, 3rd, Allweiler S, Bryan JN, Cantwell S, et al. Integrative veterinary medical education and consensus guidelines for an integrative veterinary medicine curriculum within veterinary colleges. Open Vet J. 2016;6(1):44-56. doi: 10.4314/ovj.v6i1.7
24. Stanossek I, Wehrend A. Naturheilkunde und Komplementärmedizin in der -Kleintiermedizin-–Definitionen- und- Inhalte. Tierärztliche Praxis Ausgabe K Kleintiere Heimtiere 2021. 2021;49:206-10. doi: 10.1055/a-1480-5642
25. Tricco AC, Lillie E, Zarin W, O'Brien KK, Colquhoun H, Levac D, et al. PRISMA Extension for Scoping Reviews (PRISMA-ScR): Checklist and Explanation. Ann Intern Med. 2018;169(7):467-73. doi: 10.7326/m18-0850
