# Supplementary material for: CIMUVET-survey: Complementary and Integrative Medicine (CIM) use in veterinary practice in Austria and CIM education at universities in Austria, Germany and Switzerland
Source: PLoS One. 2025 Jul 2;20(7):e0327599. doi: 10.1371/journal.pone.0327599 (PMC12221077; doi:10.1371/journal.pone.0327599)
Supplement: S2A Appendix — Overview of university courses in veterinary medicine in Austria, Germany and Switzerland. (PDF) [file pone.0327599.s003.pdf]

Supplement 2.A.

Overview of university courses in veterinary medicine in Austria, Germany and Switzerland

Tab. 1: University teaching programme in veterinary medicine in Austria
Sub-disciplines of complementary/integrative medicine according to Ng et al. 2022

	Veterinary medicine degree programme		Postgraduate education	
**University**	**Course offerings for students**	**Type of course**	**Course offerings for veterinarians**	**Type of course**
**Vetmeduni Vienna**<br>Animal nutrition and functional phytochemicals<br>Head Univ.-Prof. Dr. sc. agr. Qendrim Zebeli<br><br>Centre for Systemic Transformation and Sustainability in Veterinary Medicine<br>Head Ass. Prof. Dr. sc. agr. Barbara Metzler-Zebeli	Animal nutrition and feed science	Lecture (VO) and Exercise (UE) in the curriculum	Curriculum in veterinary phytotherapy	Further training for the Austrian Veterinary Chamber (ÖTK) diploma in veterinary phytotherapy
	Botany	Lecture (VO) and Conversation (KV) in the curriculum		
	Feeding control + dietetics in ruminants (WDK)	Elective		
	Introduction to veterinary physical medicine and rehabilitation	Elective		
	Introduction to acupuncture and neural therapy	Elective		
	Phytotherapy	Elective		
	Production and potentisation of homeopathic medicines with excursion	Elective<br>(not offered in the winter semester 2024/25)		

	https://www.vetmeduni.ac.at/fileadmin/v/z/lehre/Curricula/20220623_Curr_DS_Veterin%C3%A4rmedizin.pdf<br>https://www.vetmeduni.ac.at/fileadmin/v/z/lehre/Curricula/20220623_Curr_DS_Veterin%C3%A4rmedizin.pdf<br>https://online.vu-wien.ac.at/VUWonline/pl/ui/$ctx/wbLv.wbShowLVDetail?pStpSpNr=74572&pSpracheNr=1<br>https://online.vu-wien.ac.at/VUWonline/pl/ui/$ctx/wbLv.wbShowLVDetail?pStpSpNr=74843&pSpracheNr=1<br>https://online.vu-wien.ac.at/VUWonline/pl/ui/$ctx/wbLv.wbShowLVDetail?pStpSpNr=71182&pSpracheNr=1<br>https://online.vu-wien.ac.at/VUWonline/pl/ui/$ctx/wbLv.wbShowLVDetail?pStpSpNr=74578&pSpracheNr=1<br>https://online.vu-wien.ac.at/VUWonline/pl/ui/$ctx/wbLv.wbShowLVDetail?pStpSpNr=74889<br>https://online.vu-wien.ac.at/VUWonline/pl/ui/$ctx/wbLv.wbShowLVDetail?pStpSpNr=72468&pSpracheNr=1<br>https://www.vetmeduni.ac.at/tierernaehrung/ueber-uns/leitung<br>https://www.vetmeduni.ac.at/systemtransformation-und-nachhaltigkeit/ueber-uns	https://www.vetmeduni.ac.at/veterinaer-phytotherapie

Tab. 2: University teaching programme in veterinary medicine in Germany
The universities were sorted by location (first letter of the city name).
Sub-disciplines of complementary/integrative medicine according to Ng et al. 2022.

	Veterinary medicine degree programme		Postgraduate education	
**University**	**Course offerings for students**	**Type of course**	**Course offerings for veterinarians**	**Type of course**
**Freie Universität Berlin**<br>Centre for Animal Nutrition<br>Head Prof. Dr. med. vet. Jürgen Zentek	Animal nutrition & feed science	VO and UE integrated into the curriculum		
	Botany of feed, poisonous and medicinal plants	VO		
	https://www.vetmed.fu-berlin.de/studium/veterinaermedizin/gesetze-ordnungen/index.html<br>https://www.fu-berlin.de/vv/de/modul?id=46675&sm=851413<br>https://www.fu-berlin.de/vv/de/lv/833360?m=125157&pc=46675&sm=814672<br>https://www.vetmed.fu-berlin.de/einrichtungen/zfg/we04/Mitarbeitende/zentek_juergen/index.html<br>https://www.gesetze-im-internet.de/tappv/BJNR182700006.html			
**Justus-Liebig-Universität Gießen**<br>Institute for Animal Nutrition and Nutritional Physiology<br>Head Prof. Dr. med. vet. Klaus Eder	Animal nutrition & feed science	VO and UE integrated in the curriculum		
	Homeopathy, phytotherapy, anthroposophic medicine	VO ‘General Pharmacology and Toxicology’		
	Physiotherapy	VO		
	Botany of feed, poisonous and medicinal plants	VO		
	https://www.uni-giessen.de/de/fbz/fb10/studium-und-prufungen/studium/ECTS			

	https://www.uni-giessen.de/de/fbz/zentren/zne/mitglieder/eder<br>https://www.gesetze-im-internet.de/tappv/BJNR182700006.html			
**Tierärztliche Hochschule Hannover**<br>Institute for Animal Nutrition<br>Head Prof. Dr. med. vet. Christian Visscher	Animal nutrition, feed science, dietetics	VO and UE integrated in the curriculum and elective subjects		
	Botany of feed, poisonous and medicinal plants	VO		
	https://www.tiho-hannover.de/kliniken-institute/institute/institut-fuer-tierernaehrung/lehre<br>https://www.tiho-hannover.de/suche?L=0&id=72&tx_solr%5Bq%5D=Christian+Visscher<br>https://www.gesetze-im-internet.de/tappv/BJNR182700006.html			
**Universität Leipzig**<br>Institute for Animal Nutrition, Nutritional Damage and Dietetics<br>Head Prof. Dr. med. vet. Mirja Wilkens	Animal nutrition & feed science	VO and UE integrated in curriculum		
	Botany of feed, poisonous and medicinal plants	VO and UE integrated in curriculum		
	No information available on elective and optional subjects			
	https://www.vetmed.uni-leipzig.de/studium/ordnungen-und-leitfaeden<br>https://www.uni-leipzig.de/personenprofil/mitarbeiter/prof-dr-mirja-wilkens<br>https://www.gesetze-im-internet.de/tappv/BJNR182700006.html			
**Ludwig-Maximilians-Universität München**	Animal nutrition & feed science	VO & UE integrated into the curriculum and elective subjects	Animal nutrition and dietetics	Supplement ‘Nutritional advice for small animals’

Animal Nutrition and Dietetics<br>Head Univ.-Prof. med.vet. Nadine Paßlack	Botany of feed, poisonous and medicinal plants	VO	Animal nutrition and dietetics	Further training to become a Diplomate of the European College
	https://www.vetmed.uni-muenchen.de/studium/studienfuehrer/index.html<br>https://www.ernaehrung.vetmed.uni-muenchen.de/studium_lehre/vorlesung/index.html<br>https://www.ernaehrung.vetmed.uni-muenchen.de/kontakt/personen/index.html<br>https://www.gesetze-im-internet.de/tappv/BJNR182700006.html		https://www.ernaehrung.vetmed.uni-muenchen.de/fortbildung/index.html	

Tab. 3: University teaching programme in veterinary medicine in Switzerland
The universities were sorted by location (first letter of the city name).
Sub-disciplines of complementary/integrative medicine according to Ng et al. 2022.

	Veterinary medicine degree programme		Postgraduate education	
University	Course offerings for students	Type of course	Course offerings for veterinarians	Type of course
**Vetsuisse Fakultät Universität Bern**	Complementary medicine and specialised animal nutrition	Integrated into the clinical topics module of the Master's degree curriculum		
	General animal nutrition	Integrated into the functional basics II module of the Bachelor's degree curriculum		
	https://www.vetsuisse.unibe.ch<br>https://www.vetsuisse.unibe.ch/studium/studienaufbau/index_ger.html<br>https://www.vetsuisse.unibe.ch/studium/reglemente/index_ger.html<br>chrome-extension://efaidnbmnnnibpcajpcglclefindmkaj/<br>https://www.vetsuisse.unibe.ch/unibe/portal/fak_vetmedizin/content/e1020930/e1102593/e1244689/2024-07-10_SPVetmed_Anhaenge_de_nachVS-Fakultaetsversammlung_ger.pdf			
**Vetsuisse Fakultät Universität Zürich**	Complementary medicine and specialised animal nutrition	Integrated into the clinical topics module of the Master's degree curriculum		
	General animal nutrition	Integrated into the functional basics II module of the Bachelor's degree curriculum		

	https://www.uzh.ch/cmsssl/de/studies/programs/master/veterinary_medicine.html<br>www.vet.uzh.ch/de/studium/vetmed/Studienreglement.html<br>chrome-extension://efaidnbmnnnibpcajpcglclefindmkaj/https://www.vetsuisse.unibe.ch/unibe/portal/fak_vetmedizin/content/e1020930/e1102593/e1244689/2024-07-10_SPVetmed_Anhaenge_de_nachVS-Fakultaetsversammlung_ger.pdf	
