# Supplementary material for: CIMUVET-survey: Complementary and Integrative Medicine (CIM) use in veterinary practice in Austria and CIM education at universities in Austria, Germany and Switzerland
Source: PLoS One. 2025 Jul 2;20(7):e0327599. doi: 10.1371/journal.pone.0327599 (PMC12221077; doi:10.1371/journal.pone.0327599)
Supplement: S2B Appendix — Overview of university courses in human medicine in Austria, Germany and Switzerland. (PDF) [file pone.0327599.s004.pdf]

Supplement 2.B.

Overview of university courses in human medicine in Austria, Germany and Switzerland

Tab. 1: University courses in human medicine in Austria

The universities were sorted alphabetically based on location (first letter of the city name). The order is as follows: 1. presence of complementary/integrative medicine chairs, 2. the university includes an institute or centre for complementary/integrative medicine, 3. range of electives in the field of complementary/integrative medicine. Multiple classifications based on fulfilment of several criteria were avoided and classifications were always based on fulfilment of the most stringent criterion. When complementary/integrative medicine is mentioned, this is also understood to include sub-disciplines.

	**Medicine degree programme**		**Postgraduate education**	
1. Universities with a chair of complementary/integrative medicine*	**Course offerings for students**	**Type of course**	**Course offerings for physicians**	**Type of course**
**Sigmund Freud Privatuniversität Wien**	Complementary medicine	Integrated into the curriculum of the master's programme: human medicine & dentistry		
Chair of Complementary Medicine with a focus on Traditional Chinese Medicine (TCM) Univ.-Prof. Dr. med. univ. Tadeusz Peter Panhofer, MSc, MBA	https://med.sfu.ac.at/de/fakultaet/lehrende-an-der-fakultaet-fuer-medizin/ https://www.sfu.ac.at/wp-content/uploads/240122-MHMED23-Curriculum-Master-Humanmedizin-inkl.-Faecherliste-inkl.-ECTS.pdf			

2. Universities with a professorship of complementary/integrative medicine*				
**Paracelsus Medizinischen Privatuniversität Salzburg**<br>University Institute of Physical Medicine and Rehabilitation<br>Head<br>Prof. Dr. med. Christoph Schulze	Physical therapy & rehabilitation	Lecture (VO) and Exercise (UE) in the curriculum		
	https://www.pmu.ac.at/fileadmin/CONTENT/STUDIEN/Humanmedizin/Dokumente/240801_SPO_Humanmedizin_Diplom_REV07_published.pdf<br>https://salk.at/1725.html			
3. Universities offering courses in complementary/integrative medicine*				
**Medizinische Universität Graz**	Medicinal plants and other forms of therapy in TCM – Introduction	Specialised study module		
	neural therapy and regulatory medicine	Specialised study module		
	https://www.medunigraz.at/humanmedizin			
**Donauuniversität Fakultät für Gesundheit und Medizin – Krems**	Osteopathy	Separate independent study – Master of Science	Traditional chinese health care	University course
	Chiropractic	Separate independent study – Master of Science		
	https://www.donau-uni.ac.at/de/studium/osteopathie.html<br>https://www.donau-uni.ac.at/de/studium/chiropraktik.html		https://www.donau-uni.ac.at/dam/jcr:be574cc9-40d5-4e50-bd83-f6a4eeffc8d4/Curriculum-Traditionelle%20Chinesische%20Gesundheitspflege%20(AE)-MB-2019-92.pdf	

**Karl Landsteiner Privatuniversität Krems**	Basics of general medicine – Beyond curative medicine: Rehabilitation/Palliation in General medicine	Course in the curriculum of the 3rd semester	Therapeutic options of evidence-based phytotherapy	Certificate course
			Holistic nutritional medicine – functional medicine	Planned certificate course
	https://www.kl.ac.at/de/studium-und-weiterbildung/medizinstudium/bachelorstudium-medical-science/curriculum		https://www.kl.ac.at/de/phytotherapie<br>https://www.kl.ac.at/de/weiterbildung	
**Johannes-Kepler-Universität Linz**	Complementary medicine integrated into the general medicine module	VO		
	https://studienhandbuch.jku.at/texte/1007_7_MS_Humanmedizin.pdf<br>https://studienhandbuch.jku.at/94737<br>https://www.jku.at/studium/studienangebot/medizin/			
**Medizinische Universität Wien**	Complementary medicine: esotericism & evidence	Seminar	TCM – Basics and Practice<br>· Acupuncture for health prevention<br>· Acupuncture treatment & moxibustion<br>· Experimental Acupuncture<br>· Classical literature of acupuncture and moxibustion	Postgraduate university course (no longer offered in 2024)

	Acupuncture – what Traditional Chinese Medicine (TCM) says and what the evidence says — Seminar	
	Acupuncture in gynaecology and obstetrics — VO	
	https://campus.meduniwien.ac.at/med.campus/ee/ui/ca2/app/desktop/#/slc.tm.cp/student/courses?$ctx=&$skip=0&objTermId=144&orgId=1&q=komplement%C3%A4rmedizin<br>https://campus.meduniwien.ac.at/med.campus/ee/ui/ca2/app/desktop/#/slc.tm.cp/student/courses?$ctx=&$skip=0&objTermId=144&orgId=1&q=akupunktur	https://www.meduniwien.ac.at/web/studium-weiterbildung/universitaere-weiterbildung/alle-lehrgaenge-und-kurse/grundlagen-und-praxis-der-traditionellen-chinesischen-medizin-tcm/
4. Universities without complementary/integrative medicine courses*		
**Medizinische Universität Innsbruck**<br>(in co-operation with Schloss Hofen – Wissenschafts- und Weiterbildungs-Ges.m.b.H)		Phytotherapy — Postgraduate diploma course of the Austrian Medical Chamber (ÖAK)
		Accompanying cancer treatments — Postgraduate diploma course of the Austrian Medical Chamber (ÖAK)
		https://www.schlosshofen.at/bildung/gesundheit/phytotherapie-oeaek/<br>https://www.schlosshofen.at/bildung/gesundheit/begleitende-krebsbehandlungen-oeaek/

Tab. 2: University courses in human medicine in Germany

The universities were sorted alphabetically by location (first letter of the city name). The order is as follows: 1. presence of complementary/integrative medicine chairs, 2. the university includes an institute or centre for complementary/integrative medicine, 3. optional subjects offered in the field of complementary/integrative medicine, and 4. complementary/integrative medicine cross-sectional areas. Multiple classifications based on the fulfilment of several criteria were avoided and classifications were always based on the fulfilment of the most stringent criterion. When complementary/integrative medicine is mentioned, this is also understood to include sub-disciplines.

	**Medicine degree programme**		**Postgraduate education**	
1. Universities with a chair of complementary/integrative medicine*	**Course offerings for students**	**Type of course**	**Course offerings for students**	**Type of course**
**Universität Duisburg-Essen**<br>Chair of Naturopathy and Integrative Medicine<br>Univ.-Prof. Dr. med. Gustav Dobos	Rehabilitation, physical medicine and naturopathy	Cross-sectional area (QB) 12 in the curriculum	Naturopathy	Further training – Additional title
	For further courses, see https://www.egms.de/static/en/journals/zma/2022-39/zma001537.shtml		Integrative medicine in oncology	Further training
			Regulative and vitalizing acupuncture	Further training
			Mind-Body-Medicine	Further training
Chair of Integrative Medicine – with a focus on translational gastroenterology<br>Univ.-Prof. Dr. med. Jost Langhorst<br>at the Clinic for Integrative Medicine and Naturopathy, Bamberg Social Foundation	Introduction to integrative medicine and naturopathy internal medicine<br>Naturopathy	Elective<br><br>Practical year at the Bamberg Clinic		
	https://uk-essen.cloud.opencampus.net/sites/default/files/9-26_22aeo.pdf		https://www.nhk-fortbildungen.de/fortbildung/integrative-medizin-in-der-onkologie	

University Hospital Essen of the University of Duisburg-Essen<br>Prof. Dr. med. Tycho Zuzak	https://uk-essen.cloud.opencampus.net/de/Wahlfaecher_Klinik<br>https://www.uni-due.de/person/14537<br>https://campus.uni-due.de/lsf/rds?state=wsearchv&search=2&veranstaltung.veranstid=408169<br>https://www.uni-due.de/person/search=Jost+Langhorst?search=Jost+Langhorst<br>https://www.sozialstiftung-bamberg.de/integrativemedizin/<br>https://www.med.fau.de/studium/medizin/praktisches-jahr/lehrkrankenhaeuser/#collapse_39<br>https://kinderklinik3.uk-essen.de/haematologie-onkologie/		https://www.nhk-fortbildungen.de/fortbildung/naturheilkunde<br>https://www.nhk-fortbildungen.de/fortbildung/rava-ohrakupunktur-1-1<br>https://www.nhk-fortbildungen.de/fortbildung/mind-body-medizin
**Universität Rostock**<br>Centre for Internal Medicine<br><br>Chair of Naturopathy<br>(Univ.-Prof. Dr. med. Karin Kraft)	Rehabilitation, physical medicine and naturopathy	QB 12 in the curriculum	
	For further courses, see https://www.egms.de/static/en/journals/zma/2022-39/zma001537.shtml		
	https://naturheilkunde.med.uni-rostock.de/ueber-uns/lebenslauf-prof-dr-med-habil-karin-kraft<br>https://www.med.uni-rostock.de/fileadmin/Verwaltung/studiendekanat/humanmedizin/downloads/studienordnung_mef.pdf		
**Universitätsklinikum Tübingen**<br>Institute of General Practice and Interprofessional Care<br>Chair of Research into Complementary Medical Procedures<br>Univ.-Prof. Dr. med. Holger Cramer	Rehabilitation, physical medicine and naturopathy	QB 12 in the curriculum	
	Applied complementary and integrative medicine	Optional compulsory subject	
	Drug therapy safety	Optional compulsory module 5 integrative medicine in the master's programme	

Head<br>Prof. Dr. med. Stefanie Joos<br><br>Academic Centre for Complementary and Integrative Medicine	For further courses, see https://www.egms.de/static/en/journals/zma/2022-39/zma001537.shtml		
	https://www.medizin.uni-tuebingen.de/de/das-klinikum/einrichtungen/institute/allgemeinmedizin/lehre/wahlpflichtfach-allgemeinmedizin<br>https://www.medizin.uni-tuebingen.de/de/das-klinikum/mitarbeiter/profil/4887<br>https://www.medizin.uni-tuebingen.de/de/das-klinikum/einrichtungen/institute/allgemeinmedizin/forschung/forschungsprojekte/akademisches-zentrum-komplementaere-integrative-medizin<br>https://www.medizin.uni-tuebingen.de/de/das-klinikum/mitarbeiter/profil/1578<br>http://www.azkim.de/wer-wir-sind/		
**Universität Witten/Herdecke**<br>Institute for Integrative Medicine<br><br>Gerhard Kienle Chair of Medical Theory, Integrative and Anthroposophic Medicine<br>Univ.-Prof. Dr. med. David Martin<br><br>Institute for Integrative Healthcare and Health Promotion (IGVF)<br><br>Chair of Integrative Health Care and Health Promotion<br>Univ.-Prof. Dr. med. Tobias Esch	Anthroposophical medicine	Part-time studies	
	TCM	QB12 in the curriculum	
	Homeopathy	QB12 in the curriculum	
	Prevention, health promotion	QB10 in the curriculum	
	General practice, mind-body medicine	Practical year	
	For further courses, see https://www.egms.de/static/en/journals/zma/2022-39/zma001537.shtml		

Professorship of Paediatric and Adolescent Medicine<br>Prof. Dr. med. Alfred Längler with deputy Prof. Dr. med. Tycho Zuzak<br><br>Professorship for Training, Further Education and Continuing Education in Anthroposophic Medicine<br>Prof. Dr. med. Friedrich Edelhäuser<br><br>Professorship of Research Methodology and Information Systems in Integrative Medicine<br>Prof. Dr. med. Ekkehart Jenetzky<br><br>Gerhard Kienle Chair with Prof. Dr. sc. nat. Stephan Baumgartner, Prof. Dr.-Ing. Stanley Mungwe<br><br>Professorship for Integrative Neuromedical Sciences – with a focus on Anthroposophical Medicine<br>Prof. Dr. med. Wolfram Scharbrodt<br><br>Endowed Professorship for Rhythm Research<br>Prof. Dr. rer. nat. Dirk Cysarz	https://www.uni-wh.de/studium/studiengaenge/modellstudiengang-medizin-staatsexamen/<br>https://ibam.uni-wh.de<br>https://www.uniambulanz-witten.de/<br>https://www.uni-wh.de/ansprechpartner/friedrich-edelhaeuser<br>https://www.uni-wh.de/ansprechpartner/alfred-laengler<br>https://www.gemeinschaftskrankenhaus.de/medizin-therapie-pflege/fachabteilungen/kinder-/jugendmedizin/team/<br>https://www.uni-wh.de/gesundheit/department-fuer-humanmedizin/lehrstuehle-institute-und-zentren/institut-fuer-integrative-medizin-ifim/<br>https://gkls.uni-wh.de/personen/index.html<br>https://www.rhythmen.de/<br>https://www.uni-wh.de/ansprechpartner/peter-heusser	

Professorship in Medical Anthropology<br>Prof. Dr. med. Peter Heusser		
2. Universities with professorships in complementary/integrative medicine*		
**Charité Berlin**<br>**Institut für Sozialmedizin, Epidemiologie & Gesundheitsökonomie**<br>Professorship in Naturopathy<br>Prof. Dr. med. Benno Brinkhaus<br><br>Endowed Professorship in Integrative and Anthroposophic Medicine<br>Prof. Dr. med. Harald Matthes<br><br>Endowed Professorship in Naturopathy<br>Prof. Dr. med. Andreas Michalsen<br><br>Head of the Integrative Medicine and Digital Health working group<br>Prof. Dr. med. Claudia M. Witt, MBA<br><br>Paediatric Clinic with a focus on Oncology and Haematology<br>AG Seifert – Prevention, Integrative Medicine and Health Promotion in Paediatrics<br>Prof. Dr. med. Georg Seifert	Rehabilitation, physical medicine and naturopathy — QB 12 in the curriculum<br><br>Integrative Medicine — Optional compulsory subject<br><br>For further courses, see https://www.egms.de/static/en/journals/zma/2022-39/zma001537.shtml<br><br>https://www.charite.de/service/person/person/address_detail/univ_prof_dr_med_benno_brinkhaus/<br>https://epidemiologie.charite.de/metas/person/person/address_detail/prof_dr_med_harald_matthes/<br>https://epidemiologie.charite.de/metas/person/person/address_detail/prof_dr_med_andreas_michalsen/<br>https://epidemiologie.charite.de/metas/person/person/address_detail/prof_dr_med_claudia_m_witt_mba/<br>https://kinderonkologie.charite.de/forschung/ag_seifert/team/<br>https://campusnet.charite.de/studiengaenge/humanmedizin/themenwahl_und_wahlpflichtmodule/	

**Universitätsklinikum Freiburg**<br>University Centre for Naturopathy<br>Head<br>Prof. Dr. med. Roman Huber<br><br>Clinic for Dermatology and Venerology<br>Prof. Dr. med. Dipl. Biol. Christoph M. Schempp, Prof. Dr. rer. nat. Ute Wölfle<br><br>Academic Centre for Complementary and Integrative Medicine	Rehabilitation, physical medicine and naturopathy	QB 12 in the curriculum	Acupuncture	Further training – Additional qualification in acupuncture
	Naturopathy	Clinical elective		
	For further courses, see https://www.egms.de/static/en/journals/zma/2022-39/zma001537.shtml			
	https://www.uniklinik-freiburg.de/naturheilkunde/lehre-weiterbildung.html<br>https://www.uniklinik-freiburg.de/hautklinik/profilgruppe-entzuendliche-hauterkrankungen/forschungszentrum-skinitial.html<br>http://www.azkim.de/wer-wir-sind/		https://www.uniklinik-freiburg.de/naturheilkunde/lehre-weiterbildung.html	
**Universitätsklinikum Hamburg-Eppendorf**<br>University Cancer Centre Hamburg<br><br>Endowed Professorship for Complementary Medicine in Oncology<br>Prof. Dr. med. Matthias Rostock	Rehabilitation, physical medicine and naturopathy	QB 12 in the curriculum	Complementary medicine in oncology	Further training
	Physical and rehabilitative medicine	Elective		
	For further courses, see https://www.egms.de/static/en/journals/zma/2022-39/zma001537.shtml			
	https://www.uke.de/suchergebnisseite/index.html?q=querschnittsbereich&t=0#evo-resultsPage<br>https://www.uke.de/studium-lehre/modellstudiengang-medizin-imed/praktisches-jahr/index.html<br>https://www.uke.de/allgemein/arztprofile-und-wissenschaftlerprofile/arztprofilseite_matthias_rostock.html		2024.02.07_Flyer_Fortbildung Komplementärmedizin.indd	

**Medizinische Hochschule Hannover**<br>Klaus Bahlsen Centre for Integrative Oncology<br>Head<br>Prof. Dr. med. Diana Steinmann	Rehabilitation, physical medicine and naturopathy	QB 12 in the curriculum		
	Nutritional medicine	Elective		
	Integrative oncology	Elective		
	https://www.mhh.de/klaus-bahlsen-zentrum<br>https://www.mhh.de/medizinstudium/studienjahre			
**Universität Heidelberg**<br>University Hospital Heidelberg<br>Institute of Immunology<br>Section Molecular Immunology<br>Head<br>Prof. Dr. med. Yvonne Samstag	Rehabilitation, physical medicine and naturopathy	QB 12 in the curriculum	Clarifying molecular mechanisms of action as a foundation for evidence-based complementary and integrative medicine	Doctoral programme
Academic Centre for Complementary and Integrative Medicine	Complementary medicine with a focus on oncology care	Optional compulsory subject		
	For further courses, see https://www.egms.de/static/en/journals/zma/2022-39/zma001537.shtml			
	https://www.medizinische-fakultaet-hd.uni-heidelberg.de/fileadmin/medizinische_fakultaet/Studiendekanat/Studium/Studiengang_Humanmedizin/Heicumed_Studienordnung_03-02-7a-05.pdf<br>https://www.medizinische-fakultaet-hd.uni-heidelberg.de/studium-lehre/studium/interprofessionelle-gesundheitsversorgung-b-sc/studienverlauf-inhalte/abiturienten<br>http://www.azkim.de/wer-wir-sind/		http://www.azkim.de/aus-und-weiterbildung/promotionskolleg/	
**Universität Mainz**<br>Clinic and Polyclinic for Urology and Paediatric Urology	Rehabilitation, physical medicine and naturopathy	QB 12 in the curriculum		

Natural Products Centre<br><br>Head<br>Prof. Dr. phil. nat. Roman Blaheta	https://www.unimedizin-mainz.de/allgemeinmedizin/allgemeinmedizin/fuer-studierende/lehrangebot-uebersicht.html?L=0<br>https://www.unimedizin-mainz.de/urologie/startseite/mitarbeiter/forschung-und-lehre.html	
**Universitätsklinikum Ulm**<br>Institute for Experimental and Clinical Pharmacology, Toxicology and Naturopathy<br><br>Professorship for Experimental Pharmacology and Naturopathy<br>Prof. Dr. rer. nat. Christoph Schmidt with<br>Prof. Dr. rer. nat. Tatiana Syrovets<br><br>General and Visceral Surgery<br>Integrative Medicine Division<br>Head<br>Prof. Dr. med. Klaus Kramer<br><br>Academic Centre for Complementary and Integrative Medicine	For further courses, see https://www.egms.de/static/en/journals/zma/2022-39/zma001537.shtml<br><br>https://www.uniklinik-ulm.de/pharmakologie-toxikologie.html<br>https://www.uniklinik-ulm.de/pharmakologie-toxikologie/team.html<br>https://www.uniklinik-ulm.de/allgemein-und-viszeralchirurgie/forschung-studien/fachbereich-integrative-medizine.html<br>http://www.azkim.de/wer-wir-sind/	
**Martin-Luther-Universität Halle-Wittenberg**<br>Institute of Rehabilitation Medicine<br>Head<br>Prof. Dr. phil. Thorsten Meyer-Feil	Rehabilitation, physical medicine and naturopathy QB 12 in the curriculum<br><br>https://www.umh.de/lehre/studiengaenge/medizin<br>https://www.umh.de/einrichtungen/institute/rehabilitationsmedizin/team	

3. Universities offering a complementary/integrative medicine elective*		
**Ruhr-Universität Bochum**	Rehabilitation, physical medicine and naturopathy — QB 12 in the curriculum	
	Homeopathy — Clinical elective	
	Physical & rehabilitative medicine — Clinical elective	
	Physical therapy – naturopathy — Clinical elective	
	https://medizinstudium.ruhr-uni-bochum.de/medidek/infoszumstudium/amtliches/AB-1279.pdf https://medizinstudium.ruhr-uni-bochum.de/medidek/infoszumstudium/wahlfaecher/Index.cfm	
**Heinrich-Heine-Universität Düsseldorf** Institute for Transplantation Diagnostics and Cell Therapeutics	Rehabilitation, physical medicine and naturopathy — QB 12 in the curriculum	
	https://www.medizinstudium.hhu.de/fileadmin/redaktion/Fakultaeten/Medizinische_Fakultaet/Medizinstudium/Dokumente/Ordnungen_und_Regularien/Modellstudiengang/Studien-_und_Pruefungsordnung_fuer_den_Modellstudiengang.pdf	
**Universitätsklinikum Düsseldorf der Heinrich-Heine-Universität Düsseldorf** Institute of General Medicine	Ear acupuncture — Elective	
	Naturopathy and complementary medicine — Elective	

	Phytotherapeutics and ethnopharmacology	Elective	
	History and development of homeopathy	Elective	
	https://www.medizinstudium.hhu.de/duesseldorfer-curriculum-medizin/wahlcurriculum https://www.medizinstudium.hhu.de/fileadmin/redaktion/Fakultaeten/Medizinische_Fakultaet/Medizinstudium/Dokumente/Wahlcurriculum/Wahlcurriculum_Faecheruebersicht.pdf		
**Universität Lübeck**	Rehabilitation, physical medicine and naturopathy	QB 12 in the curriculum	
	For further courses, see https://www.egms.de/static/en/journals/zma/2022-39/zma001537.shtml		
	https://www.uni-luebeck.de/studium/studiengaenge/humanmedizin/studieren-lehren/klinik.html		
**Otto von Guericke Universität Magdeburg** Institute of General Medicine	Rehabilitation, physical medicine and naturopathy including classical naturopathy, mindfulness, self-care, mind-body medicine, theory and practice of classical homeopathy, fundamentals of TCM, introduction to anthroposophic medicine	QB 12 in the curriculum	
	Homeopathy	Clinical elective	
	https://ialm.med.ovgu.de/Lehre.html		
**Ludwig-Maximilians-Universität München**	Rehabilitation, physical medicine and naturopathy	QB 12 in the curriculum	

	Diagnostics and acupuncture	Compulsory elective seminar	
	Homeopathy	Elective	
	Basics of homeopathy and their critical examination	Elective	
	General medicine – naturopathy	Elective	
	https://www.mecum.med.uni-muenchen.de/studium/klinik/lnw_klinik.pdf<br>https://www.mecum.med.uni-muenchen.de/index.html<br>https://lsf.verwaltung.uni-muenchen.de/qisserver/rds?state=wsearchv&search=1&subdir=veranstaltung&veranstaltung.dtxt=Hom%C3%B6opathie&veranstaltung.semester=20242&P_start=0&P_anzahl=12&P.sort=&_form=display<br>https://lsf.verwaltung.uni-muenchen.de/qisserver/rds?state=wsearchv&search=1&subdir=veranstaltung&veranstaltung.dtxt=naturheilverfahren&veranstaltung.semester=20242&P_start=0&P_anzahl=12&P.sort=&_form=display		
**Friedrich-Alexander-Universität Erlangen Nürnberg**	Rehabilitation, physical medicine and naturopathy	QB 12 in the curriculum	
	Introduction to integrative medicine and naturopathy	Optional compulsory subject at the Bamberg Clinic	
	https://www.doc.zuv.fau.de//L1/PO/Med/Medizin_ER<br>BT_Staatsexamen/konsolidierte_Fassungen/StuPO		

	_Medizin_und%20Medizin_ERBT_StuPOMed_20191009.pdf<br>https://www.sozialstiftung-bamberg.de/integrativemedizin/		
**Julius-Maximilians-Universität Würzburg**	Rehabilitation, physical medicine and naturopathy	QB 12 in the curriculum	
	Physical therapy	Clinical elective	
	Behavioural therapy	Clinical elective	
	https://www.uni-wuerzburg.de/studium/angebot/faecher/medizin/		
	https://www.med.uni-wuerzburg.de/fileadmin/03000000/user_upload/dateien_studiendekanat/stundenplaene_wahlfaecher/Wahlfaecher_Seminar_und_UaK__Klinik_SS_2024_Stand__110924.pdf		
4. Universities offering complementary/integrative medicine as a cross-sectional area*			
**RWTH Aachen University**	Rehabilitation, physical medicine and naturopathy	Course in the 10th semester – compulsory course	
	https://www.medizin.rwth-aachen.de/cms/medizin/die-fakultaet/institute-und-kliniken/die-institute/klinisch-theoretische-institute/institut-fuer-pharmakologie-und-toxikolo/~dpxf/lehre/		
**Universität Bielefeld**	Rehabilitation, physical medicine and naturopathy	QB 12 in the curriculum	
	https://ekvv.uni-bielefeld.de/sinfo/publ/staatspruefung/medizin/pdf;jsessionid=619B4812B59337AFA28CE2FDDFC433E0		

**Universität Bonn**	Rehabilitation, physical medicine and naturopathy	QB 12 in the curriculum	
	https://www.medfak.uni-bonn.de/de/studium-lehre/beratung-und-service/humanmedizin/klinik/mo-klinik-humanmedizin/files/studien-und-einteilungsplaene/studienplan_5_sose24.pdf		
**Medizinische Hochschule Brandenburg Theodor Fontane**	Rehabilitation, physical medicine and naturopathy	QB 12 in the curriculum	
	https://www.mhb-fontane.de/de/medizin-studieren chrome-extension://efaidnbmnnnibpcajpcglclefindmkaj/https://www.mhb-fontane.de/files/Dateien/studiengang-humanmedizin/studienordnung-bmm-2022.pdf		
**Universität Dresden**	Rehabilitation, physical medicine and naturopathy	QB 12 in the curriculum	
	https://tu-dresden.de/studium/vor-dem-studium/studienangebot/sins/sins_studiengang?autoid=309		
**Goethe Universität Frankfurt am Main**	Rehabilitation	QB 12 in the curriculum	
	https://qis.server.uni-frankfurt.de/qisserver/rds?state=wtree&search=1&trex=step&root120241=104732%7C104735%7C105572%7C105805%7C102613&P.vx=kurz		
**Universität Gießen**	Rehabilitation, physical medicine and naturopathy	QB 12 in the curriculum	
	https://www.uni-giessen.de/de/mug/6/pdf/6_60_11_2_ANL2_1ae		

**Universitätsmedizin Göttingen**	Rehabilitation, physical medicine and naturopathy QB 12 in the curriculum<br>https://www.umg.eu/studium-lehre/studieren-an-der-umg/studiengaenge/humanmedizin/klinik-humanmedizin/module/m42-erkrankungen-der-bewegungsorgane-einschliesslich-rheumatischer-erkrankungen-und-trauma/	
**Universität Greifswald**	Rehabilitation, physical medicine and naturopathy QB 12 in the curriculum<br>https://www.uni-greifswald.de/storages/uni-greifswald/2_Studium/2.4_Rund_um_die_Pruefungen/2.4.1_Pruefungs_und_Studienordnungen/Staatsexamen/Humanmedizin/PSO_Humanmedizin_2019.pdf	
**Friedrich-Schiller-Universität Jena, Thüringen**	Rehabilitation, physical medicine and naturopathy QB 12 in the curriculum<br>https://www.uniklinikum-jena.de/studiendekanat/Studien_+und+Pr%C3%BCfungsamt/Humanmedizin/Zweiter+Abschnitt/Lehrveranstaltungen.html	
**Christian-Albrechts-Universität zu Kiel**	Rehabilitation, physical medicine and naturopathy QB 12 in the curriculum<br>https://www.studium.uni-kiel.de/de/pruefungen/andere/andere#M	
**Universität Köln**	Rehabilitation, physical medicine and naturopathy QB 12 in the curriculum<br>https://medfak.uni-koeln.de/studium-lehre/studiengaenge/humanmedizin/klinik	

**Universität Leipzig**	Rehabilitation, physical medicine and naturopathy	QB 12 in the curriculum	
	https://www.uniklinikum-leipzig.de/studium-lehre/studium/humanmedizin https://www.uniklinikum-leipzig.de/einrichtungen/isap/Seiten/Lehre/Querschnittsbereiche-(QSB).aspx		
**Universitätsklinikum Mannheim der Universität Heidelberg**	Accidents, rehabilitation, physical medicine and naturopathy	QB 12 in the curriculum	
	https://www.umm.uni-heidelberg.de/studium/		
**Philipps Universität Marburg**	Rehabilitation, physical medicine and naturopathy	QB 12 in the curriculum	
	https://marvin.uni-marburg.de/qisserver/pages/cm/exa/coursecatalog/showCourseCatalog.xhtml?_flowId=showCourseCatalog-flow&_flowExecutionKey=e1s3		
**Universität Münster**	Rehabilitation, physical medicine	QB 12 in the curriculum	
	https://medicampus.uni-muenster.de/kl_qbereiche0.html		
**Universität Regensburg**	Rehabilitation, physical medicine and naturopathy	QB 12 in the curriculum	
	https://www.uni-regensburg.de/chemie-pharmazie/pharmakologie-toxikologie/ag-haen/lehre/index.html		
**Universität des Saarlandes**	Rehabilitation, physical medicine and naturopathy	QB 12 in the curriculum	

	https://www.uni-saarland.de/fakultaet-m/lehre/medizin/zweiter-studienabschnitt/uebersicht.html	

Tab. 3: University courses in human medicine in Switzerland

The universities were sorted alphabetically based on location (first letter of city name). The order is as follows: 1. presence of complementary/integrative medicine chairs, 2. the university includes an institute or centre for complementary/integrative medicine, 3. optional

subjects offered in the field of complementary/integrative medicine. Multiple assignments based on the fulfilment of several criteria were avoided and assignments were always made based on the fulfilment of the highest criterion. When complementary/integrative medicine is mentioned, this also includes sub-disciplines.

	**Medicine degree programme**		**Postgraduate education**	
1. Universities with a chair complementary/integrative medicine*	**Course offerings for students**	**Type of course**	**Course offerings for students**	**Art der Lehrveranstaltung**
**Universität Bern**<br>Institute for Complementary and Integrative Medicine<br>Chair for Complementary and Integrative Medicine<br>Univ.-Prof. Dr. med. Ursula Wolf	Complementary medicine in human medicine	VO<br>Elective internships<br>Advanced seminar<br>Internship		
	https://www.ikim.unibe.ch/studium/lehrveranstaltungen/index_ger.html<br>https://www.medizin.unibe.ch/ueber_uns/personen/prof_dr_wolf_ursula/index_ger.html			
**Universität Zürich**<br>Institute for Complementary and Integrative Medicine<br>Chair for Complementary and Integrative Medicine<br>Univ.-Prof. Dr. med. Claudia Witt	Complementary medical procedures – principles, application and evaluation	*Compulsory elective module of the bachelor's programme*		
	Chiropractic medicine	Separate bachelor's programme (curriculum as for human medicine plus additional courses with a focus on chiropractic)		
	Chiropractic medicine	Master's mono study programme		
	https://www.usz.ch/fachbereich/komplementaere-und-integrative-medizin/<br>https://studentservices.uzh.ch/uzh/anonym/vvz/index.html?sap-language=DE&sap-ui-language=DE#/SC/2024/003/50000976			

	https://www.usz.ch/team/claudia-witt/		
2. Universities with professorships in complementary/integrative medicine*			
**Universität Basel**<br>Translational Complementary Medicine<br>Head<br>Prof. Dr. Carsten Gründemann	Introduction to complementary medicine	VO - Bachelor's programme	
	Skin – treating external symptoms internally	VO - Bachelor's programme	
	General oncology	VO - Bachelor's programme	
	Management of side effects of cancer	VO - Bachelor's programme	
	Antibiotic-free and other treatments for lung infections	VO - Bachelor's programme	
	Acupuncture and hypnosis	VO - Master's programme	
	Integrative medicine	VO - Master's programme	
	Pharmacokinetics, drug interactions	VO - Master's programme	
	Translational complementary medicine	Research seminar	

	https://medizinstudium.unibas.ch/fileadmin/user_upload/medizin/Dokumente/Lehrkonzept/2024_01_19_LC_Komplementaermedizin.pdf https://vorlesungsverzeichnis.unibas.ch/de/home?keyword=komplement%C3%A4rmedizin	
**Universität Freiburg** Institute of Primary Care Prof. Dr. med. Pierre-Yves Rodondi	Complementary medicine — Clinical basic subjects - Bachelor's programme	
	https://cdn.unifr.ch/scimed/plans/current/Plan_BMed_de.pdf https://www.unifr.ch/med/de/section/staff/all/people/9127/2db72	
**Universität Lausanne** Centre de médecine intégrative et complémentaire (Prof. Dr. med. Chantal Berna Renella)	Integrative and complementary medicine — VO advanced seminar	
	https://www.unil.ch/central/en/home.html https://applicationspub.unil.ch/interpub/noauth/php/Un/UnPers.php?PerNum=50374&LanCode=37&menu=ens	
3. Universities offering courses in complementary/integrative medicine*		
**Universität Genf**	Website in French only	
	https://www.unige.ch/medecine/enseignement1/bachelor-et-master-en-medecine-humaine	
